# Segmental morphometrics of the olive baboon (*Papio anubis*): a longitudinal study from birth to adulthood

**DOI:** 10.1111/joa.12602

**Published:** 2017-03-14

**Authors:** François Druelle, Peter Aerts, Kristiaan D'Août, Valérie Moulin, Gilles Berillon

**Affiliations:** ^1^ Laboratory for Functional MorphologyBiology DepartmentUniversity of AntwerpAntwerpBelgium; ^2^ Département de PréhistoireMusée de l'HommeUMR 7194 CNRS‐MNHNParisFrance; ^3^ Primatology StationUPS 846 CNRSRousset‐sur‐ArcFrance; ^4^ Biomechanics and Motor Control of Human MovementDepartment of Movement and Sport SciencesUniversity of GhentGhentBelgium; ^5^ Institute of Ageing and Chronic DiseaseUniversity of LiverpoolLiverpoolUK

**Keywords:** development, inertial properties, locomotion, *Papio anubis*, primate model

## Abstract

The linear dimensions and inertial characteristics of the body are important in locomotion and they change considerably during the ontogeny of animals, including humans. This longitudinal and ontogenetic study has produced the largest dataset to date of segmental morphometrics in a Catarrhini species, the olive baboon. The objectives of the study were to quantify the changes in body linear and inertial dimensions and to explore their (theoretical) mechanical significance for locomotion. We took full‐body measurements of captive individuals at regular intervals. Altogether, 14 females and 16 males were followed over a 7‐year period, i.e. from infancy to adulthood. Our results show that individual patterns of growth are very consistent and follow the general growth pattern previously described in olive baboons. Furthermore, we obtained similar growth curve structures for segment lengths and masses, although the respective time scales were slightly different. The most significant changes in body morphometrics occurred during the first 2 years of life and concerned the distal parts of the body. Females and males were similar in size and shape at birth. The rate and duration of growth produced substantial size‐related differences throughout ontogeny, while body shapes remained very similar between the sexes. We also observed significant age‐related variations in limb composition, with a proximal shift of the centre of mass within the limbs, mainly due to changes in mass distribution and in the length of distal segments. Finally, we observed what we hypothesize to be ‘early biomechanical optimization’ of the limbs for quadrupedal walking. This is due to a high degree of convergence between the limbs’ natural pendular periods in infants, which may facilitate the onset of quadrupedal walking. Furthermore, the mechanical significance of the morphological changes observed in growing baboons may be related to changing functional demands with the onset of autonomous (quadrupedal) locomotion. From a wider perspective, these data provide unique insights into questions surrounding both the processes of locomotor development in primates and how these processes might evolve.

## Introduction

The locomotor apparatus of animals can be considered best adapted morphologically when its size and shape are well suited to the principal mode of locomotion of the species (see Witte et al. 1991; Preuschoft & Günther, 1994; Preuschoft et al. 1996). Primates are of special interest because, unlike many mammals with more specialized modes of locomotion, they adopt a variety of positional modes every day (Hunt et al. 1996) that involve a diverse range of substrate reaction forces (in terms of magnitude and direction) and moments. This is easily understood in view of the complex three‐dimensional environment in which they evolved. Their locomotor apparatus should thus reflect, in some respects, an adaptive trade‐off between efficiency, stability (static and dynamic), manoeuvrability and flexibility of movement.

It has been hypothesized that adaptations of the body to specific locomotor modes are likely to become more pronounced with development (Isler et al. 2006). This would make differential growth an important process for the locomotor transitions observed during primate ontogeny (e.g. Doran, 1997; Dunbar & Badam, 1998; Wells & Turnquist, 2001; Sarringhaus et al. 2014). Newborn primates are unable to walk: it commonly takes several weeks to months (depending on the species) to achieve this with a semblance of interlimb coordination, and from months to years to become fully efficient and autonomous. Their locomotor development is affected by complex processes involving different factors such as neuro‐muscular maturation, experience and changes in body dimensions (e.g. Nakano, 1996; Adolph & Avolio, 2000; Garwicz et al. 2009; Kimura & Yaguramaki, 2009; Adolph et al. 2012). Adult catarrhine primates, including humans and their closest relatives, exhibit an impressive diversity in their morphometrics (i.e. linear dimensions, segmental masses, moment of inertia) in parallel with a wide diversity of locomotor modes. Yet these species appear to follow a common pattern of differential growth (Leigh et al. 2009). Consequently, studying the development of the locomotor apparatus in full is crucial to a better understanding of the ontogeny of locomotion potentially leading to specialized modes or generalized repertoires. Furthermore, such studies could improve our knowledge about the evolvability of locomotor profiles, i.e. ‘the ability of random variations to sometimes produce improvement’ (Wagner & Altenberg, 1996).

This paper focuses on changes in morphometrics and their (theoretical) mechanical significance during the ontogeny of the catarrhine olive baboon, *Papio anubis,* based on a longitudinal study of a large sample of captive individuals. At the adult stage, baboons are specialized for quadrupedal locomotion, yet they are also capable of adopting a variety of positional modes, including bipedalism (e.g. Rose, 1977). Just after birth, baboons only possess grasping abilities, but it has been shown that growing infants rapidly include quadrupedal walking and suspensory, climbing and leaping behaviour in their repertoire. As a result, infants exhibit a more diverse repertoire than adults (Druelle & Berillon, 2013). Furthermore, according to previous behavioural and biomechanical observations (Altmann & Samuels, 1992; Raichlen, 2005a; Druelle et al. in press), an autonomous locomotor profile is established in the first year of life, during which the functional demands on the limbs change in parallel with significant morphological changes. Such modifications in size and shape seem to adapt the body optimally for quadrupedal walking (Raichlen, 2005a). However, potential predisposing traits in the intrinsic morpho‐dynamic properties of the limbs (i.e. those morphometrics that determine the intrinsic dynamics of the limb segments) may also favour early quadrupedal locomotion (Druelle et al. in press). In adult baboons, it has been demonstrated that there is a divergence in mass distribution between the forelimbs and hindlimbs that creates a natural pendular period (NPP) convergence (Raichlen, 2004). It has been previously argued that exhibiting good NPP convergence between the four limbs is likely to minimize muscle activity, and therefore energy expenditure, during quadrupedal locomotion (Myers & Steudel, 1997; Raichlen, 2004). It has further been hypothesized that the divergence in limb mass distribution might reflect the need to maintain precise grasping abilities in the hands in order to forage successfully after infants become less reliant on gripping the mother with the feet for transport (Raichlen, 2005b). In this context, we have produced the largest dataset of segmental morphometrics for a Catarrhini species to date and made it available for further exploration, e.g. in biomechanical studies such as (inverse) dynamic analyses of movement or musculoskeletal modelling. Based on this dataset and given that mass in newborn baboons is distally concentrated into the fore‐ and hindlimbs and that the body centre of mass has a more cranial position, we first hypothesized that, during early life, the morphology must adjust rapidly to meet the mechanical demands of developing the locomotor repertoire. It should be noted that in baboons, this repertoire tends to be quadrupedal, but that other locomotor abilities are also present. Nevertheless, according to our recent study on the early development of quadrupedal walking in baboons (Druelle et al. in press), the NPP of the four limbs should be strongly convergent initially. Adult baboons are sexually dimorphic (Leigh et al. 2009) and sex‐related differences have been observed in the timing and intensity of their growth patterns (Glassman et al. 1983; Coelho, 1985; Glassman & Coelho, 1987; Leigh et al. 2009) and in their biomechanics (see hindlimb compliance in Patel et al. 2013). With regard to morphometrics, we hypothesized that females would have a lower rate of mass gain and that this pattern would end earlier than in males.

## Materials and methods

### Study site and subjects

This longitudinal study was conducted at the CNRS Primatology Station (UPS 846) at Rousset‐sur‐Arc, France, where approximately 300 baboons are bred and housed. From this population, we regularly measured 14 females and 16 males from infancy (minimum age 2 months) to adulthood; this experiment was conducted over a period of 7 years (Letter of approval MP/01/15/02/08 from the Midi‐Pyrénées Region Ethics Committee, February 2008). The individuals were measured every 3 months until they were 2 years old and then every 6 months. All the individuals studied are part of the same troop and live together in a large park with various enrichments (Anvari et al. 2014; Berillon et al. 2010; Druelle & Berillon, 2013; see Supporting Information Table S1 for information on the individuals).

### Collecting morphometrics

Our measurement protocol allows the use of the geometric model developed by Crompton et al. (1996), as previously applied to baboons (Raichlen, 2004, 2005b; Druelle et al. 2016a). External linear measurements of the nine body segments were taken (head, including the neck, trunk, upper arm, forearm, hand, thigh, shank, foot and tail). The landmarks used are the same as in the previous studies (see Appendix in Schoonaert et al. 2007). The animals were anaesthetized to take the measurements (maximum duration, 20 min), and by convention, the limbs were measured on the left side only in order to ensure consistency between successive sessions and individuals, as well as to limit the duration of the anaesthesia. We obtained individual segment dimensions (lengths and antero‐posterior and medio‐lateral diameters) and estimated the inertial properties of the body segments from the model:

- segment mass, using segment average densities obtained from cadavers (Berillon et al. pers. obs.);
- segment centre of mass (CoM), calculated from the proximal joint of the segment (from the hip joint for the trunk segment);
- segment mass moment of inertia in the sagittal (*I*_*x*_) and coronal (*I*_*y*_) planes; the third moment of inertia in the horizontal plane was not calculated for this study because of its low impact on locomotor mechanics (cf. Schoonaert et al. 2007). Note that the moment of inertia about the segment CoM can be find using the parallel axis theorem (see Crompton et al. 1996; Raichlen, 2004).

We calculated inertial parameters for the whole body and the whole forelimb and hindlimb. Using the mathematical and geometric software geogebra 5.0, we designed free body diagrams of baboons at two extreme stages of their development, separately for males and females. We used a typical body posture (quadrupedal standing) observed in the wild with an average elbow joint angle of 155° and knee joint angle of 138° for all morphotypes (see Patel et al. 2013). We calculated the position of the whole body centre of mass (BCoM) in this posture, as well as the fore‐ and hindlimb centre of mass (CoM1 and CoM2, respectively). The CoM position was estimated as follows (Miller et al. 1973):

$$\text{CoM}=\frac{\sum_{i=1}^{n}m_{i}r_{i}}{\sum_{i=1}^{n}m_{i}}$$

where *m*_*i*_ is the mass of one segment and *r*_*i*_ is the distance of the segment CoM from the joint of origin (e.g. hip or shoulder). We then calculated the radius of gyration of the whole limb (RG) in the straight position. RG is a measure of overall limb mass distribution; it represents the distance of a point mass from the proximal joint that would give an equivalent inertia to the original limb. This is expressed as the square root of the whole limb's mass moment of inertia about the proximal joint (which may be the shoulder or the hip), divided by the mass of the limb considered:

$$\text{RG}=\sqrt{\frac{I}{M}}$$

where *I* is the whole limb's mass moment of inertia about the proximal joint (calculated using the parallel axis theorem; see Raichlen, 2004), *M* is the mass of the whole limb. RG can be normalized via a percentage of limb length. The natural pendular period (NPP) is a good way to represent the limb's intrinsic morpho‐dynamics (Myers & Steudel, 1997; Raichlen, 2004; Druelle et al. in press). This is calculated as follows:

$$\text{NPP}=2\pi \sqrt{\frac{I}{M\times g\times \text{CoM}}}$$

where *g* is the gravitational constant (9.8 m s^−2^). We calculated the NPP of the fore‐ and hindlimbs for extended limbs with the hand and foot in a straight line with other segments. This procedure enables comparisons with previous studies (Myers & Steudel, 1997; Raichlen, 2004).

### Statistics

In accordance with Crompton et al. (1996) and Raichlen (2004), the reliability of the external measurements procedure was assessed by calculating an estimated total body mass via the sum of the segment masses per individual. This estimated mass (*y*) was compared with that directly measured with an electronic scale (*x*) yielding an absolute error calculation:

$$\frac{\left|x-y\right|}{x}\times 100$$

To model individual growth patterns, we regressed each individual's absolute segmental morphometrics (length, mass, *I*_*x*_, *I*_*y*_) against the age at the time of measurement. Because each variable is age‐dependent, we used type 1 regression models and their reliability was tested using the Fisher test. The slope and intercept values obtained for the significant models were used to test whether individual growth patterns followed the general pattern of growth, i.e. when all longitudinal data are grouped together. After controlling for the normality of the sample of individual growth patterns (Shapiro–Wilks test), we applied the one sample *t*‐test to assess whether the mean of the sample of individual growth patterns was equal to the mean of the general growth pattern. This analysis was done using R 2.15 software (The R Foundation, Vienna, Austria, https://www.rproject.org/). To test differences between sexes, we used the slope values and the intercept values of the individual models. Comparisons were performed with permutation tests for independent samples (staxact 3.1 software, Cytel, Inc., Cambridge, MA, USA).

**Figure 1:**
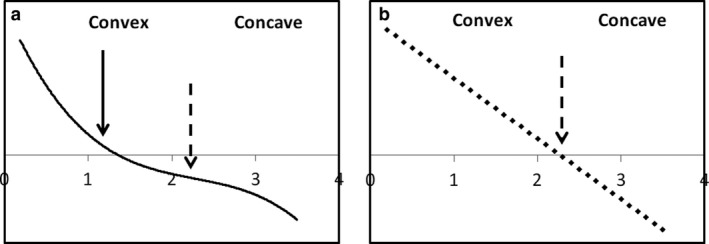
(a) Original curve, (b) curve obtained with the second derivation. The dashed arrow indicates the point of inflection of the original curve and the solid arrow indicates the median, which is used to determine the period of time during which the variation of the original curve changes, i.e. decreasing in this example.

Major age‐related changes in segment lengths and masses were assessed using principal component analyses (PCA) following Druelle et al. (2016a). The set of variables was made dimensionless before being entered into the PCA: the length variables were divided by the cube root of the total body mass measured and the segment masses were divided by the total estimated body mass (Hof, 1996; Raichlen, 2005b). First, the PCA allowed us to reduce the number of variables. Secondly, it allowed us to determine the most significant changes occurring in the body morphometrics during growth. Thirdly, we plotted the principal component [the interpretability of the first eigenvalue was assessed using the broken‐stick method (Frontier, 1976)] against age and we used a polynomial of degree *n* with a least‐squares fit in order accurately to describe the period in which the most significant changes occur; linear models were not reliable here because of the autocorrelation of the distribution of residuals. The best fitting model is obtained when the coefficient of determination is considered high, i.e. *R* ^2^ > 0.5, and higher degrees do not further improve the fit (note that the end of the curve is likely to be biased because of the end of the dataset, which is why we have indicated this part with a dashed line). To estimate the developmental period when the amount of variation changes, i.e. decrease or increase in the growth rate, we calculated the second derivative of each polynomial model. A change in the sign of the second derivative curve indicates a point of inflection. The period of growth rate decrease or increase was determined from the median of the previous period, or the median between two points of inflection (see Fig. 1 for an example). We also performed a PCA, including general body morphometrics, i.e. segment lengths, segment masses and the total body mass, on 10 age–sex classes (0–6 months, 1½–2 years, 3–3½ years, 4½–5 years, 6–6½ years). This analysis was added for two reasons: first, to produce general age‐related baboon morphotypes (coupled body size and body shape) and show how these change; secondly, to describe general differences between males and females during ontogeny for a confirmation of our previous tests.

Furthermore, according to previous contributions showing the importance of the shape and size of limbs in locomotion (see Kilbourne & Hoffman, 2013), we specifically analyzed the relationships between whole fore‐ and hindlimbs in straight positions. For each variable, i.e. limb length, limb mass, limb CoM, limb moment of inertia about the proximal joint, and limb NPP, we plotted the hindlimb against the forelimb. Because the two pairs of limbs vary together with age, we modelled these relationships using the reduced major axis (RMA), which is an orthogonal regression (type 2 model). The general growth pattern was thus assessed using the identity line: if the slope of the latter falls inside the confidence interval (95%) of the slope of the RMA model, this indicates a similar (isometric) growth pattern between fore‐ and hindlimbs. As we did for the full dataset, we validated the general growth pattern using comparisons with the mean of the sample of individual patterns (one sample *t*‐test after controlling for normality of the sample). In addition, we plotted the dimensionless CoM and the dimensionless RG (CoM and RG are normalized via a percentage of limb length) against age and used the polynomial method previously described in order to assess the transitions. The significance level is 0.05 for all tests.

## Results

### Validity of the geometric model

The total body mass estimated from the direct external measurements is very close to that measured on a scale. We obtained, for females, a mean absolute error of 4.98 ± 1.69% (mean ± standard deviation) per individual measured and 5.16 ± 1.3% for males. These values strongly validate the reliability of the procedure and confirm the accuracy of the geometric model when applied to baboons; it was also validated previously on cadavers of different primate species using double‐pendulum experiments (Crompton et al. 1996; Raichlen, 2004). This geometric model makes it possible to perform *in vivo* analyses of morphological parameters, thus offering new opportunities such as longitudinal follow up and observation, at the individual level, of performance–morphology interactions (Raichlen, 2005a, 2006; Young et al. 2007; Raichlen et al. 2009; Van Dam et al. 2010, 2011) and behaviour–morphology interactions (Druelle et al. 2016a,b).

### Total body mass

**Figure 2:**
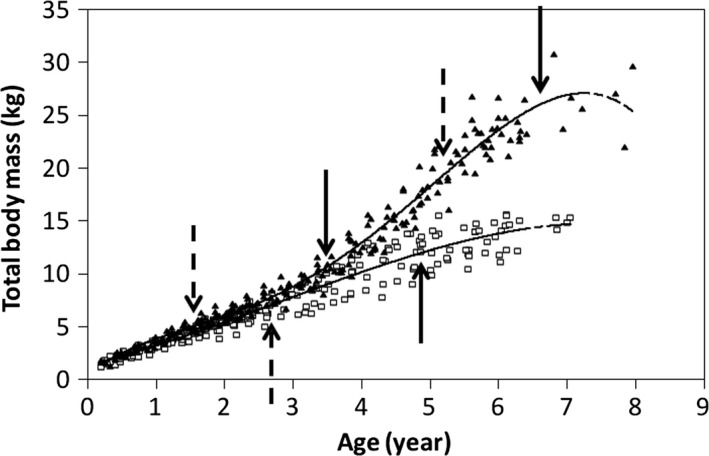
Total body mass as a function of age for 14 female baboons (white squares) and 16 male baboons (black triangles). All longitudinal data are plotted together for each sex‐class. The dashed arrow indicates the point of inflection of the original curve and the solid arrow indicates the median, which is used to determine the period of time during which the variation of the original curve changes.

Figure 2 shows that the polynomial model gives a good estimation of the increase in total body mass for female and male baboons (*R* ^2^ = 0.93 and 0.97, respectively). We observed two points of inflection for males, indicating an increase in the rate of mass gain at around 3.3 years of age and growth ending at around 6.55 years of age. In females, the general pattern is more linear than in males. We observed one point of inflection indicating a decrease in mass gain at around 4.9 years of age. This indicates the end of growth for females (Table 1).

**Table 1:** Polynomial regression models for total body mass

Sex	Polynomial equation	*R* ^2^	Point of inflection (year)	Variation in rate of change (year)
Female	−0.04x^3^+0.3x^2^+1.57x+1.39	0.93	2.75	4.9
Male	−0.04x^4^+0.52x^3^−1.83x^2^+4.61x+0.61	0.97	1.55–5.05	3.3–6.55

### Individuality within the general growth pattern

Table 2 shows the absolute segment linear and inertial parameters via the slopes (a) and intercepts (b) of the linear regression models. All the models are strongly significant (*P* < 0.0001) and illustrate the general growth pattern of the olive baboon. Individual growth patterns closely match this general pattern. In females, no significant difference was found after testing for comparisons between individual and general patterns (Supporting Information Table S2). In males, we found only one significant difference for the foot mass (Supporting Information Table S3).

**Table 2:** General growth pattern of segment inertial parameters represented by the linear regression model (*y* = a*age + *b*)

	Length (mm)		Mass (kg)		Ix (kg mm^−2^)		Iy (kg mm^−2^)	
	*a*	*b*	*a*	*b*	*a*	*b*	*a*	*b*
Females								
Head	10.75	114.72	0.18	0.29	1830.37	5.08	1849.83	−8.40
Trunk	33.58	233.79	1.10	0.46	74465.39	−45258.43	74056.72	−44859.45
Upper arm	16.02	102.67	0.08	0.01	829.34	−588.09	843.05	−600.66
Forearm	17.11	111.14	0.04	0.04	551.64	−266.72	550.84	−266.53
Hand	7.35	81.19	0.02	0.03	73.45	6.74	70.58	6.71
Thigh	15.82	108.61	0.17	0.05	1734.90	−1155.79	1793.15	−1200.92
Shank	15.62	111.39	0.05	0.03	641.62	−301.36	645.83	−305.03
Foot	11.46	118.61	0.03	0.05	222.34	29.11	217.17	27.98
Tail	20.92	291.90	0.03	0.05	957.71	365.70	957.71	365.70
*P*‐value sex‐related								
Head	< 0.0001	–	< 0.0001	–	< 0.0001	–	< 0.0001	–
Trunk	0.0001	–	< 0.0001	–	< 0.0001	–	< 0.0001	–
Upper arm	< 0.0001	–	< 0.0001	–	< 0.0001	–	< 0.0001	–
Forearm	0.0003	–	< 0.0001	–	< 0.0001	–	< 0.0001	–
Hand	0.0003	–	< 0.0001	–	< 0.0001	–	< 0.0001	–
Thigh	0.0006	–	< 0.0001	–	< 0.0001	–	< 0.0001	–
Shank	< 0.0001	–	< 0.0001	–	< 0.0001	–	< 0.0001	–
Foot	< 0.0001	–	< 0.0001	–	< 0.0001	–	< 0.0001	–
Tail	0.0001	–	< 0.0001	–	< 0.0001	–	< 0.0001	–
Males								
Head	16.98	109.49	0.37	0.03	5194.88	−5529.72	5263.64	−5621.16
Trunk	42.38	229.80	1.91	−0.81	162313.09	−193597.2	161090.04	−191720.7
Upper arm	20.92	96.94	0.14	−0.07	1925.15	−2410.45	1963.93	−2466.18
Forearm	21.24	106.16	0.08	−0.02	1233.91	−1423.89	1232.18	−1422.69
Hand	10.25	80.68	0.03	0.01	177.33	−126.63	169.77	−121.02
Thigh	20.15	102.29	0.30	−0.16	3861.46	−4835.30	4065.20	−5134.29
Shank	20.46	104.98	0.09	−0.02	1397.32	−1569.96	1408.82	−1586.85
Foot	15.79	115.45	0.05	0.02	464.58	−326.20	450.86	−316.19
Tail	34.13	278.38	0.06	0.01	2642.74	−2289.72	2642.74	−2289.72

Regarding sex‐related differences during ontogeny, it is clear that males exhibit a higher growth rate in length and mass for all the segments. The intercept values were not tested for differences because the lack of measurements at birth makes the origin of the calculated models redundant (see the negative values sometimes obtained: Table 2). However, by taking the data from the first measurement session, i.e. when the individuals were youngest [between 2 and 5 months for males (*n* = 7) and females (*n* = 7)], we were able to test early sex‐related differences. Only one significant difference was found: the length of the hand is longer in males than in females (79 and 74 mm, respectively, permutation test: −1.584, *P = *0.0061).

### Changes in morphotypes (dimensionless segment lengths and masses) with age

**Figure 3:**
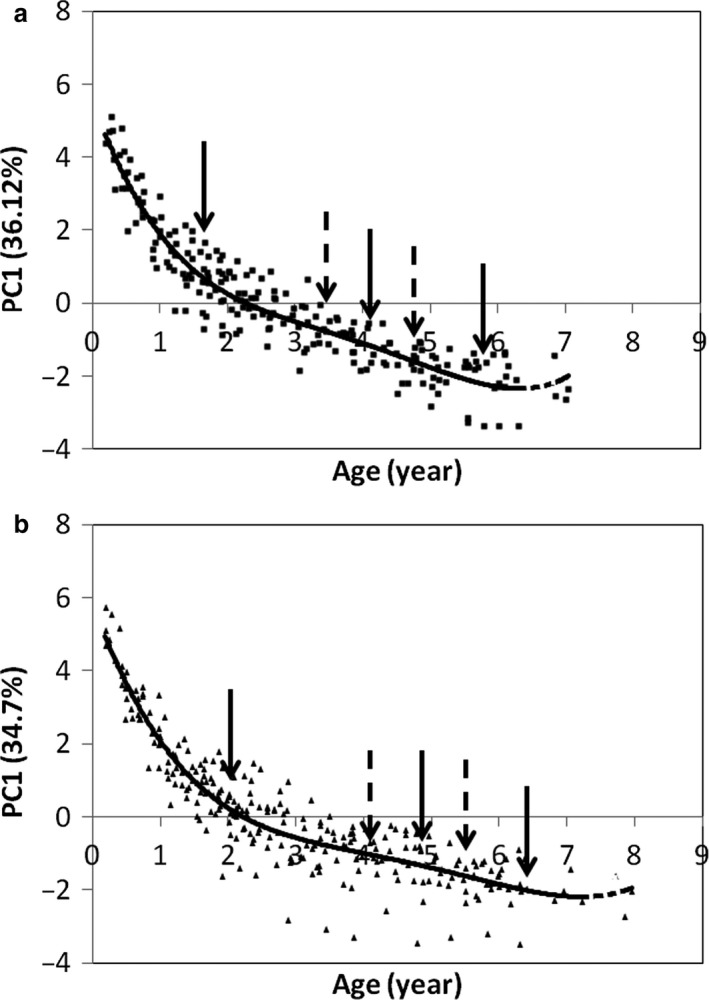
Relationship between the projection of the individuals on PC1 (relative segment lengths) and age. (a) All females and sessions plotted together; (b) all males and sessions plotted together.

The PCA produces a new principal component that explains a large fraction of the variance in segment lengths (36.12% in females and 34.7% in males) and in segment masses (46.85% in females and 47.62% in males); the first observed eigenvalues, 3.25, 3.12, 4.22 and 4.29, respectively, always exceed the first eigenvalue provided by the broken‐stick method, 2.83. Regarding segment lengths, the first dimension is compiled from the same segments for both sexes: the head [factor score (FS) female: 0.9; male: 0.86], the hand (FS female: 0.89; male: 0.84), the foot (FS female: 0.89; male: 0.84) and the tail (FS female: 0.77; male: 0.78) are positively correlated. This dimension is strongly correlated with age for females: *r* = −0.89 (*P* < 0.0001) and males: *r* = −0.83 (*P* < 0.0001); Fig. 3 illustrates these relationships. The 4th order polynomial gives a good estimate of growth‐related changes: females: *R* ^2^ = 0.9; males: *R* ^2^ = 0.86.

**Figure 4:**
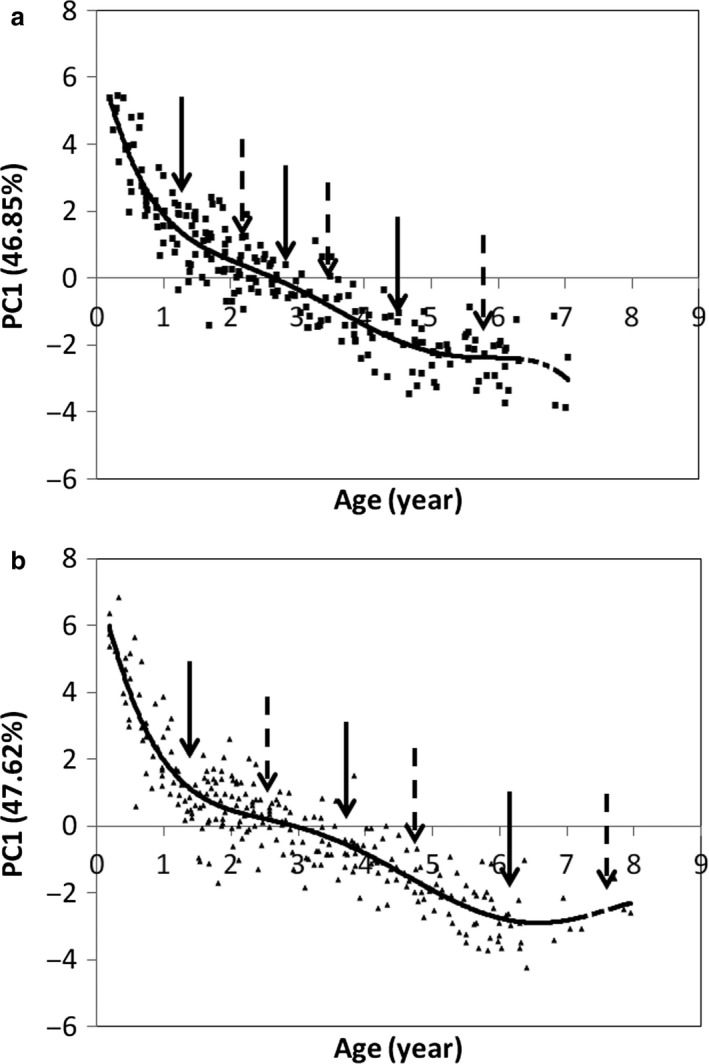
Relationship between the projection of the individuals on PC1 (relative segment masses) and age. (a) All females and sessions plotted together; (b) all males and sessions plotted together.

Regarding segment masses, the first dimension is compiled from the same segments for both sexes: the head (FS female: 0.9; male: 0.86), the hand (FS female: 0.88; male: 0.87), the foot (FS female: 0.84; male: 0.8) and the tail (FS female: 0.75; male: 0.77) are negatively correlated with trunk mass (FS female: −0.72; male: −0.78). This dimension is strongly correlated with age for females: *r* = −0.88 (*P* < 0.0001) and males: *r* = −0.86 (*P* < 0.0001); Fig. 4 illustrates these relationships. The polynomial is a good estimate of these changes: females: *R* ^2^ = 0.86 (5th order polynomial), males: *R* ^2^ = 0.85 (5th order polynomial).

Using the second derivative method (see Statistics), we were able to detect the significant transitions in the growth patterns of segment length and segment mass (Table 3).

**Table 3:** Polynomial regression models for body dimensions (dimensionless values)

Sex	Polynomial equation	*R* ^2^	Point of inflection (year)	Variation in rate of change (year)
Segment lengths				
Female	0.0187x^4^ − 0.3035x^3^ + 1.7885x^2^ − 5.16x + 5.5505	0.9	3.35–4.75	1.65–4.05–5.9
Male	0.0114x^4^ − 0.2191x^3^ + 1.5418x^2^ − 5.1015x + 5.8506	0.86	4.05–5.55	2–4.8–6.3
Segment masses				
Female	−0.0106x^5^ + 0.2079x^4^ − 1.5057x^3^ + 5.0409x^2^ − 8.7328x + 6.8916	0.86	2.25–3.55–5.95	1.1–2.9–4.75
Male	−0.0053x^5^ + 0.1313x^4^ −1.176x^3^ + 4.7429x^2^ − 9.2897x + 7.5563	0.85	2.55–4.55–7.85	1.25–3.55–6.2

#### Segment length

In females, the greatest changes occur between birth and 1.65 years (Fig. 3). The growth rate is slower between 1.65 and 4.05 years and accelerates between 4.05 and 5.9 years of age, when growth stops. In males, the greatest changes occur between birth and 2 years of age. The rate of change is slower between 2 and 4.8 years and accelerates between 4.8 and 6.3 years of age, when growth stops.

#### Segment mass

A similar pattern appears for segment masses, but the changes occur earlier (Fig. 4). In females, the greatest changes occur between birth and 1.1 years. The rate of change is slower between 1.1 and 2.9 years and accelerates between 2.9 and 4.75 years of age, when growth stops (at 4.9 years for total mass gain). In males, the greatest changes occur between birth and 1.25 years. The rate of change is slower between 1.25 and 3.55 years and accelerates between 3.55 and 6.2 years of age, when growth stops (at 6.55 years for total mass gain).

**Figure 5:**
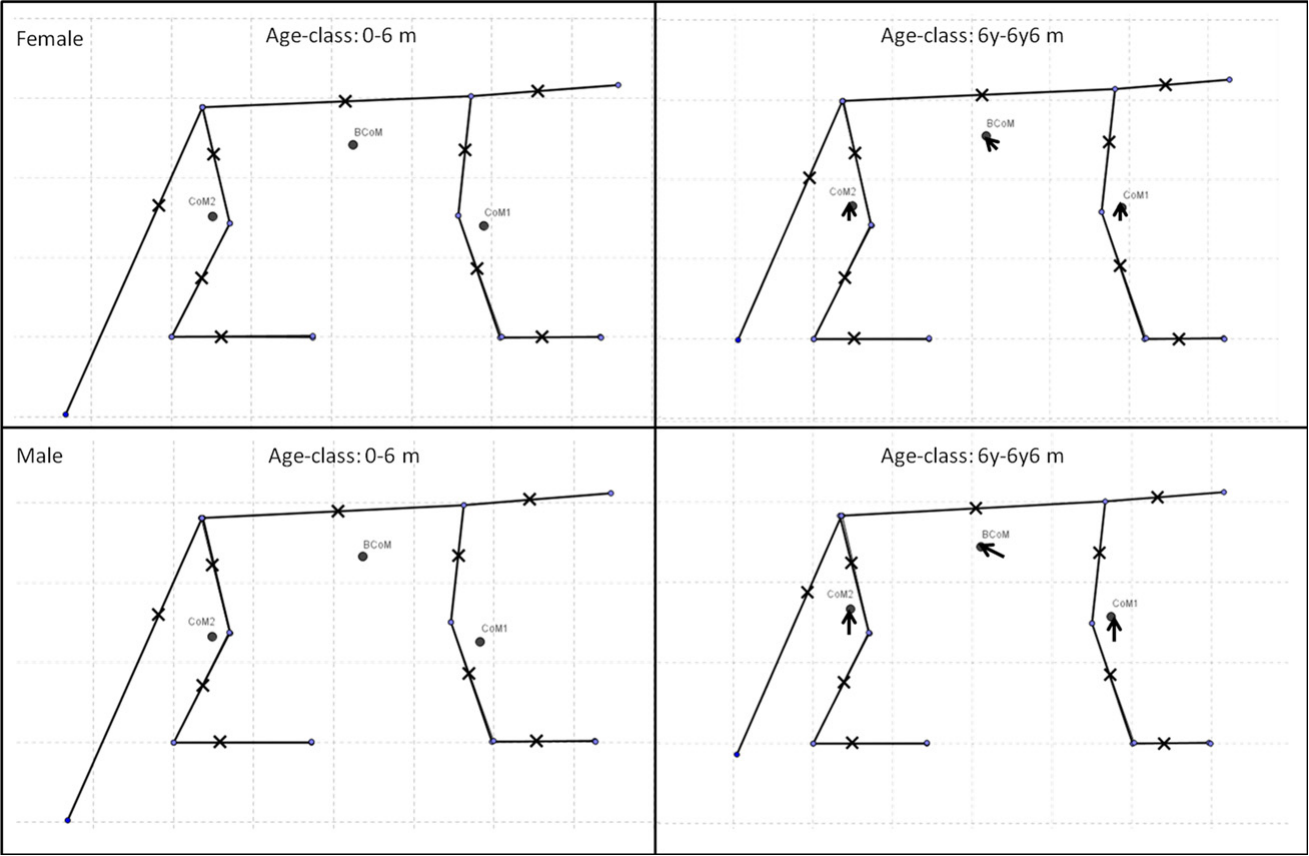
Morphotypes of female (age‐class 1, *n* = 12 and age‐class 2, *n* = 10) and male (age‐class 1, *n* = 13 and age‐class 2, *n* = 10) baboons at two extreme stages of their ontogeny. The crosses represent the segment CoM, the small dots represent the joints between segments. All segment lengths are made dimensionless via the cube root of the total body mass.

**Figure 6:**
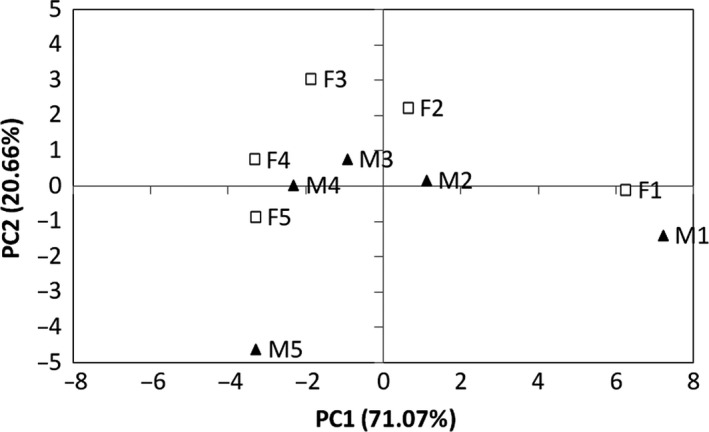
Projection of the 10 age–sex classes on the first composed plan of the PCA applied to dimensionless values (segment lengths and masses) and total body mass. Female baboons are represented with white squares and males are represented with black triangles. Numbers indicate the stage in development: 1 = 0–6 months; 2 = 1½–2 years; 3 = 3–3½ years, etc.

Based on these changes in body morphometrics, we were able to design the two extreme morphotypes of the baboon at birth and at the beginning of adulthood. Figure 5 illustrates these changes and their impact on the position of the BCoM as well as CoM1 (forelimb) and CoM2 (hindlimb). During development, we clearly observed a caudal shift of the BCoM into the trunk and a proximal shift of the CoM into the fore‐ and hindlimbs. The PCA applied to the 10 age‐related morphotypes (see Statistics) produced two newly composed principal components that explain a large fraction of the variance (91.72%; Table 4). The two eigenvalues observed exceed the eigenvalues provided by the broken‐stick method (PC1: 13.50 > 2.83, PC2: 3.92 > 1.83). Therefore, the first composed plan can be interpreted and allows general comparisons between females and males (Fig. 6). PC1 represents age‐related variations in relative segment masses (except for the shank) and shows that the most significant differences occur during the first year of life. Female morphotypes, at all stages of their development, are closer to the adult morphotype than in males. Furthermore, in females, there is no difference between morphotypes 4 and 5. According to this axis, the last two female morphotypes and the last male morphotype are very similar. PC2 represents relative limb length and total body mass. At all stages in their development, males are relatively heavier and have shorter relative limb lengths than females. In males, a significant increase in total body mass occurs in the period between morphotypes 4 and 5, which corresponds to the acceleration in mass gain described previously (Table 1); this was not found in the female growth pattern.

**Table 4:** Eigenvalues and eigenfactors of the PCA of morphotypes

	Dimension 1	Dimension 2
Eigenvalue	13.5	3.92
Variance (%)	71.07	20.66

	Factor 1	Factor 2
Relative mass		
Hand	**0.9491**	0.0935
Forearm	**0.9747**	0.0607
Upper arm	**−0.9337**	−0.2665
Thigh	**−0.9599**	−0.0151
Shank	0.5338	0.2562
Trunk	**−0.979**	0.0258
Head	**0.9816**	−0.0816
Tail	**0.9626**	0.1875
Foot	**0.9337**	0.3263
Total body mass	−0.788	**−0.5466**
Relative length		
Hand	**0.9804**	0.1355
Forearm	−0.5484	**0.7892**
Upper arm	−0.7594	**0.6081**
Thigh	−0.6194	**0.7739**
Shank	−0.3662	**0.8855**
Trunk	−0.5148	**0.7895**
Head	**0.9763**	−0.095
Tail	**0.9127**	0.3727
Foot	**0.9083**	0.4028

Numbers in bold indicate the main contributing factors.

### Whole limb inertial properties: forelimb vs. hindlimb

**Figure 7:**
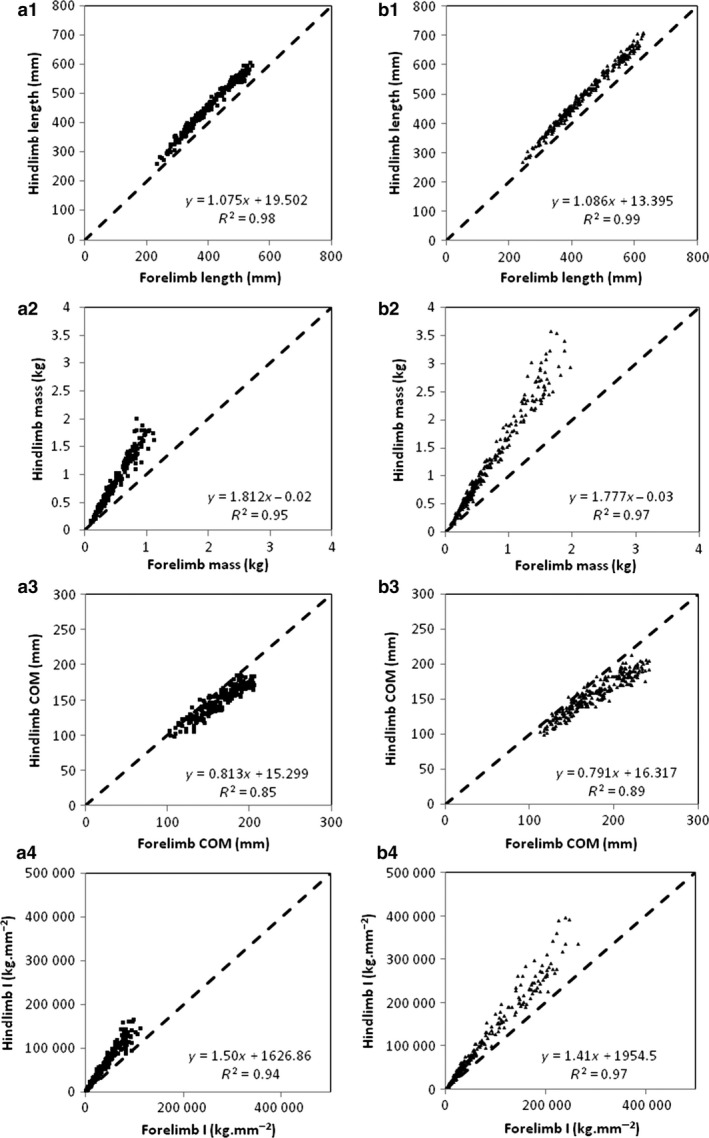
Relationship between fore‐ and hindlimb length (1), Mass (2), CoM (3) and mass moment of inertia about the proximal joint (4) in female (a) and male (b) baboons. The dashed line is the line of identity (*x *= *y*). The linear equations represent the RMA regressions calculated with the full dataset for each variable and for each sex. The coefficients of determination indicate high reliability of the linear models in describing these relationships with age.

Judging by the high values of the coefficients of determination, the limb growth pattern is well estimated by RMA models (Fig. 7). There is no difference between the general pattern of limb growth from the full dataset and the individual pattern (Supporting Information Tables S4 and S5). The general patterns of growth are similar for male and female baboons (see Figs 3 and 4). Therefore, it is possible to describe a general pattern of limb growth in baboons. During ontogeny, forelimb length, from the full dataset, is always and consistently smaller than hindlimb length [slope (95% CI): females 1.08 (1.05–1.1); males 1.09 (1.07–1.1)]. From the full dataset, the proximal shift of the CoM is more pronounced in hindlimbs than in forelimbs [slopes (95% CI): females 0.81 (0.77–0.86); males 0.79 (0.76–0.82)]. From the full dataset, the mass and moment of inertia of the hindlimbs about the proximal joint gradually increase relatively to the forelimbs [slopes (95% CI): 1.81 (1.76–1.87) and 1.78 (1.74–1.81), 1.50 (1.46–1.55) and 1.41 (1.38–1.44) for females and males, respectively].

**Figure 8:**
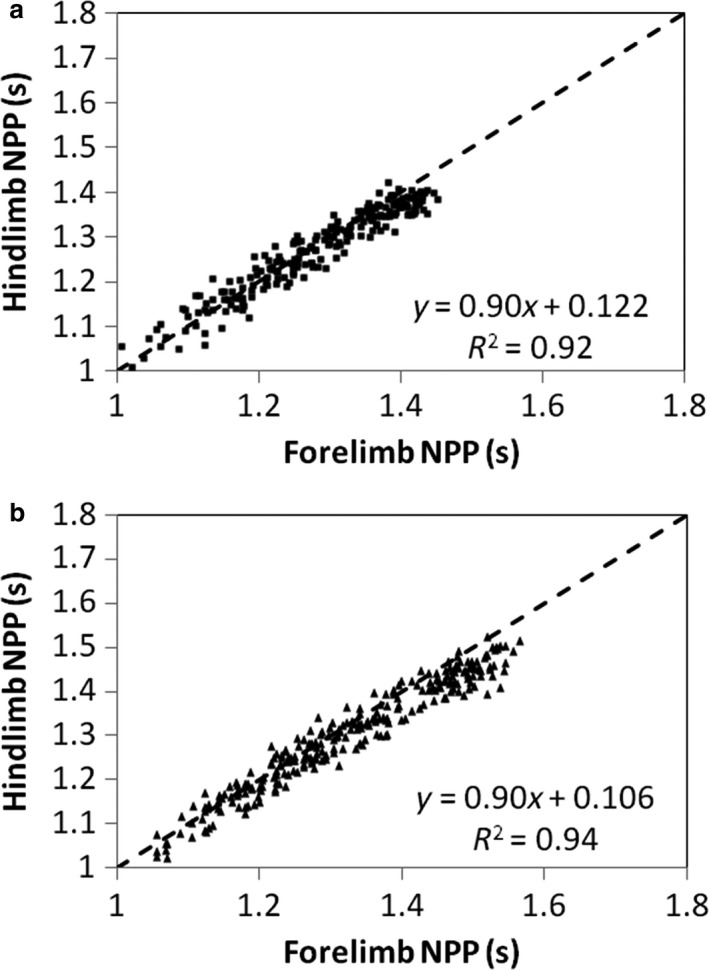
Relationship between the NPP of the hindlimb and the NPP of the forelimb in female (a) and male (b) baboons. The dashed line is the line of identity. The linear equations represent the RMA regressions calculated with the full dataset for each variable and for each sex. The coefficients of determination indicate high reliability of the linear models in describing this relationship with age.

Regarding NPP convergence, although the coupling between forelimb and hindlimb is always close during development (Fig. 8), the linear models show a trend towards a gradual increase in the forelimb NPP relatively to the hindlimb NPP [from the full dataset, slope (95% CI): 0.90 (0.86–0.93) and 0.90 (0.88–0.93) for females and males, respectively]. We split our sample into 1‐year age‐classes in order to highlight the period in which the convergence of the NPP decreases. The forelimb NPP is significantly greater than the hindlimb NPP from 3 years of age onwards in females and from 1 year of age onwards in males. Therefore, convergence is optimal in young infants (Table 5). Although a tendency towards more divergent NPPs is observed, this does not prevent very good convergence between both pairs of limbs at the adult stage as well (2.48% difference in females and 3.25% in males).

**Table 5:** Comparisons of fore‐ and hindlimb NPPs during baboon ontogeny

Sex	Age‐class	FL mean	HL mean	FL–HL abs%diff	FL–HL Paired test
Female	0–1	1.11	1.11	2.56	0.7693
	1–2	1.21	1.21	1.82	0.7250
	2–3	1.28	1.28	1.66	0.3553
	3–4	1.35	1.33	1.88	0.0008
	4–5	1.38	1.36	1.99	< 0.0001
	5–…	1.40	1.37	2.48	< 0.0001
Male	0–1	1.12	1.11	2.18	0.0578
	1–2	1.22	1.21	2.09	0.0099
	2–3	1.30	1.28	1.91	0.0022
	3–4	1.37	1.35	2.31	0.0002
	4–5	1.45	1.42	2.63	< 0.0001
	5–6	1.50	1.45	3.65	< 0.0001
	6–…	1.50	1.46	3.25	0.0001

**Figure 9:**
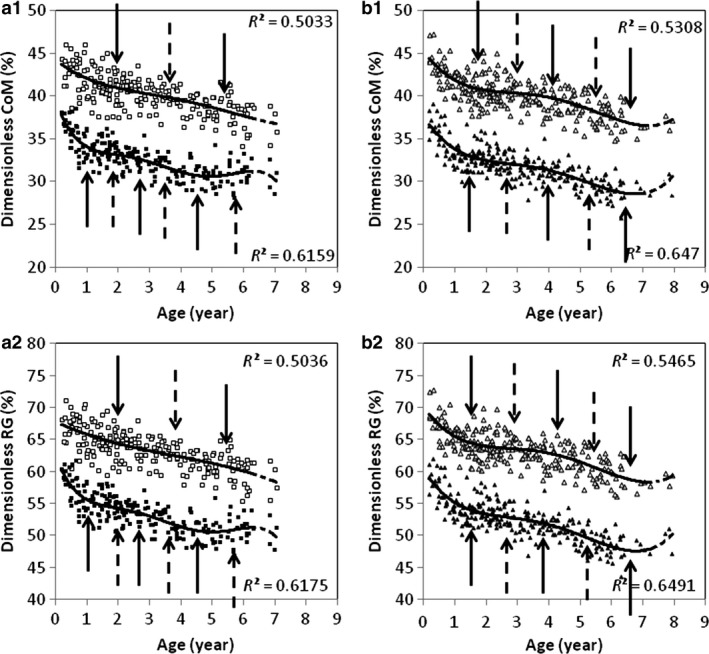
Relationship between the position of the CoM (1) and age, and the position of the RG (2) and age in female (a) and male (b) baboons. The white colour represents the forelimb, the black colour represents the hindlimb. The polynomial models are represented in the point clouds.

Polynomial models yield good estimates for age‐related variations in CoM and RG into fore‐ and hindlimbs (i.e. normalized via a percentage of limb length), as seen from the high values of the coefficients of determination (Fig. 9). Using the second derivative method (see Statistics), we determined the periods in which the CoM shifts proximally and RG changes in magnitude, towards a shorter radius, within the limbs (Table 6).

**Table 6:** Age‐related migration of the CoM and RG into the limbs

Sex	Limbs	Polynomial equation	*R* ^2^	Point of inflection (year)	Variation in rate of change (year)
Centre of mass					
F	Fore	−0.0315x^3^ + 0.3586x^2^ − 2.0254x + 43.82	0.50	3.75	1.85–5.4
	Hind	−0.0183x^5^ + 0.3393x^4^ − 2.294x^3^ + 7.014x^2^ − 10.483x + 39.504	0.62	1.95–3.45–5.75	0.95–2.7–4.6
M	Fore	0.0239x^4^ − 0.3997x^3^ + 2.2135x^2^ − 5.3761x + 45.38	0.53	2.75–5.65	1.35–4.2–6.85
	Hind	0.0277x^4^ − 0.4423x^3^ + 2.3898x^2^ − 5.8444x + 37.679	0.65	2.75–5.25	1.35–4–6.65
Radius of gyration					
F	Fore	−0.0311x^3^ + 0.356x^2^ − 2.3023x + 67.763	0.50	3.85	1.9–5.45
	Hind	−0.0232x^5^ + 0.4283x^4^ − 2.88x^3^ + 8.7323x^2^ − 13.111x + 62.431	0.62	1.95–3.45–5.75	0.95–2.7–4.6
M	Fore	0.0324x^4^ − 0.5396x^3^ + 2.9761x^2^ − 7.1829x + 70.207	0.55	2.75–5.55	1.35–4.15–6.8
	Hind	0.0381x^4^ − 0.6054x^3^ + 3.2282x^2^ − 7.8193x + 60.294	0.65	2.65–5.25	1.3–3.95–6.65

In females, three periods were plotted for the hindlimbs, and two periods for the forelimbs. In the hindlimbs, the greatest proximal shift of the CoM occurs between birth and 0.95 years. The shift continues at a lower rate from 0.95 to 2.7 years of age, then accelerates from 2.7 to 4.6 years of age, when the shift ends. In the forelimbs, the proximal shift of the CoM is close to the linear model. With the polynomial model, there is a more significant shift from birth to 1.85 years. This continues at a slightly slower rate from 1.85 to 5.4 years of age, when the shift comes to an end.

In males, three periods were plotted for hindlimbs and forelimbs. The greatest changes occur between birth and 1.35 years for both pairs of limbs. The shift continues at a slower rate from 1.35 to 4 years and 4.2 years for hindlimbs and forelimbs, respectively, then accelerates in the hindlimbs from 4 to 6.65 years of age, when the shift comes to an end. In the forelimbs, it accelerates from 4.2 to 6.85 years of age, when it ends.

As seen in Fig. 9, the mass is more proximally concentrated in the hindlimbs than in the forelimbs at all stages of development in both sexes. In both pairs of limbs, baboons experience a proximal migration of the CoM. The results for the RG follow those for the CoM.

## Discussion

### Growth pattern

The shapes of the growth curves for skeletal length and segment mass are very similar. Although the same number of phases (variation in rates of change) is observed for segment length and mass, the timescales vary. The greatest changes occur in the first phase, corresponding to infancy; the second involves a significant decrease in the rates of change, which speed up again in the third phase up to adulthood. Our first hypothesis was that newborn baboons would have distally concentrated limb masses and that a new morphological pattern would develop rapidly to meet the mechanical demands of the onset of a quadrupedally oriented locomotor profile. Our results support this hypothesis: the greatest changes in body morphometrics occurred during the first year of life for segment mass and during the first 2 years for segment length. This involves a decrease in relative lengths and masses in the distal segments, i.e. the hand, foot, head and tail. From the general morphotypes, we also observed a relative increase in proximal segment masses, i.e. the thigh and upper arm, and in the trunk, as well as a relative decrease in forearm mass; no changes occurred in relative lengths for these segments. There is therefore a proximal shift of the CoM within the limbs that appears to depend mainly on the changes in relative segment masses and lengths (for autopodia); note that the CoM into each segment does not change with age. This growth pattern may be related to changes in limb functional demands because it matches the period in which baboons gradually become more active and autonomous, mainly through play and exploratory activities (Rose, 1977). According to our results, the next developmental period involves changes at a slower growth rate. There is a third and final period during which the growth rate increases slightly up to the end of development. The observation of these different phases is consistent with the work of Leigh et al. (2009), who observed the occurrence of growth spurts in baboons.

Although there are very few ontogenetic data of this kind for Catarrhini species in general, our results can nevertheless be compared with those of Turnquist & Wells (1994) for another cercopithecoid species, the rhesus macaque, and of Schoonaert et al. (2007) for a hominoid species, the chimpanzee. As previously proposed by Leigh et al. (2009), the olive baboon's pattern of growth is similar, in some respects, to that of these two species. In macaques, the relative mass of the proximal limb segments increases while the relative masses of the distal limb segments and the head decrease. There is also a slight shift in the relative lengths of the fore‐ and hindlimbs in macaques, with the hindlimbs becoming relatively longer, whereas the slight difference in the limb lengths of baboons remains unchanged with age. Furthermore, the forearm and shank seem to remain constant throughout ontogeny in macaques, which is not the case for the relative mass of the forearm in baboons (cf. results reported here on general morphotypes). Because keeping more intrinsic muscle mass in forearms increases manual grasping abilities, this difference may be related to the fact that macaques spend more time in trees than baboons do. Similarly, the growth pattern of chimpanzees (Schoonaert et al. 2007) points to a shift from distal to proximal limb mass distribution, mainly through a decrease in the relative mass of the hand and foot and an increase in the relative mass of the thigh. There is also a significant decrease in the relative head mass but no increase in the relative trunk mass. Unlike macaques and baboons, chimpanzees have longer forelimbs than hindlimbs throughout their ontogeny. Nevertheless, the relative mass of the forearm and shank remains constant throughout ontogeny, as in macaques, and the upper arm does not increase in relative mass. This points to a need to maintain good grasping abilities in the hands in order to move efficiently in trees. Schoonaert et al. (2007) reported that the decrease in the relative mass of the foot and hand may be due to a decrease in dimensionless segment lengths with age. Our results also suggest this relationship because the reduction in the relative mass of autopodia (hands and feet) parallels the reduction in the relative length of these segments. Preuschoft (2004) suggested that the independence of the grip is ensured by short intrinsic muscles of the hands and feet: longer digits would therefore require stronger and heavier muscles. Although having relatively longer and/or heavier distal segments has been correlated with grasping ability (Jungers & Fleagle, 1980; Raichlen, 2005b; Lawler, 2006; Druelle et al. 2016b), it has also been suggested that intrinsic morphological proportions, i.e. the length of digits relative to metapodials, may be a substantial determinant of grasping performance (Lemelin & Schmitt, 2007; Young & Heard‐Booth, 2016). In addition, having relatively longer distal segments may also facilitate some locomotor tuning (e.g. Lawler, 2006) by providing a larger area of support that potentially increases stability in quadrupedal locomotion at an early age.

### Sex‐related differences

Sex‐related differences have been previously observed in the duration and rate of the growth pattern of baboons (Glassman et al. 1983; Glassman & Coelho, 1987; Leigh et al. 2009). Our large ontogenetic and longitudinal sample confirms this. All the linear models based on absolute values (segment length, mass and moment of inertia) differ between males and females, which means that most sex‐related differences can be directly attributed to the higher absolute segment lengths and masses in male baboons, also resulting in higher values for the moment of inertia. Nevertheless, comparing infant male and female baboons in early infancy revealed only one significant difference, in hand length. Therefore, just after birth, male and female baboons are very similar in shape and size. Regarding total body mass, a rapid increase begins at around 3½ years of age in males and lasts until growth ceases (at 6½ years), whereas in females no changes were observed in the rate of mass gain, and growth stops earlier, at around 5 years of age. However, when corrected for total body mass effect, we observed a very similar pattern of growth for both sexes. According to the PCAs, males and females undergo the same transitional periods with similar changes in relative segment masses and lengths. Nevertheless, these transitional periods always occur later in males than in females, who reach adult body dimensions at an earlier age: before 6 years of age for relative segment length and before 5 years of age for relative segment mass, whereas males reach adult dimensions in relative length and mass a little after 6 years of age. Regarding the proximal shift of the limb CoM, the pattern appears to be similar between sexes in the hindlimbs, but subject to different processes in the forelimbs. In females, the shift is almost linear until around 5½ years of age, while the proximal shift observed in males follows the typical pattern found for other body dimensions, i.e. with considerable changes initially followed by a decrease in the growth rate, and a final acceleration just before growth ceases.

### Development of the limbs

Our results show that the forelimbs are shorter than the hindlimbs in baboons and that this relationship is consistent throughout their development. It should be noted that the decrease in the intermembral index (without the hands and feet) observed at around 5 months of age by Shapiro & Raichlen (2006) in *Papio cynocephalus* is not marked here when observing the full duration of development, but is likely to become apparent with a shorter observation window. Three aspects have been considered: (i) the difference in shape between fore‐ and hindlimbs relatively to limb length, (ii) the impact of development on limb shape and (iii) the actual difference in shape between fore‐ and hindlimbs, i.e. relative to the proximal joint.

- Quadrupedal locomotion involves differential use of the limbs: hindlimbs are commonly used for propulsion while forelimbs are used more for braking (Demes et al. 1994). Furthermore, in primates, there is a need to maintain stronger grasping abilities in the hands than in the feet for foraging and manipulative functions (e.g. Pouydebat et al. 2014). Therefore, a disparity between mass distribution patterns in the fore‐ and hindlimb would reflect variations in the functional roles of the limbs during development (e.g. Rose, 1977; Raichlen, 2005b; Patel et al. 2013). Our results support this assumption in the sense that the CoM (linear effects) and RG (rotational effects) are always more distally positioned, relative to limb lengths, in the forelimbs than in the hindlimbs. For example, at 3½ years of age, we observed that the forelimb CoM is approximately in the same relative position as the hindlimb CoM was at birth (39.92 and 39.02%, respectively, in females; 40.24 and 38%, respectively, in males), whereas the latter is more proximally concentrated (7.09% more proximal in females and 6.29% in males).
- Our results show that during early development, infant baboons experience a proximal shift of the CoM and RG in the fore‐ and hindlimbs. Regarding the kinematics of their quadrupedal walking, it has been shown that, at a very early age, infant baboons adopt longer stride lengths and lower stride frequencies because of their distally concentrated limb mass (Raichlen, 2005a). Furthermore, they exhibit total power outputs (i.e. the sum of internal and external power) similar to other quadrupedal mammals developing a more proximally concentrated limb mass (Raichlen, 2006). Therefore, it seems that infant baboons are using a trade‐off mechanism that adapts their kinematics to reduce the energy expenditure induced by the movement of distally heavy limbs. Nevertheless, the CoM migration found in the present study is likely to reduce the rotational inertia of distal limb segments, and therefore the energy costs of swinging limbs in adults walking quadrupedally (Wickler et al. 2004). This supports the assumption that morphological adaptations become more pronounced during ontogeny.
- After birth, actual fore‐ and hindlimb CoM and RG are very similar relative to their respective proximal joints, i.e. the shoulder and hip. However, as a result of the proximal migration of the CoM described above, the actual forelimb CoM is more distally positioned than the actual hindlimb CoM at the end of development. On this point, it appears that, at an early age, differences in fore‐ and hindlimb mass distribution patterns compensate for differences in fore‐ and hindlimb lengths by creating fore‐ and hindlimb NPP convergence (see Myers & Steudel, 1997; Raichlen, 2004). With age, there is a loss of this convergence due to changes in limb composition. Although, during walking, specific mechanisms such as limb angular excursion may actively correct potential NPP divergence in adults (hindlimb excursion is relatively greater in baboons, Larson et al. 2001), it appears that swinging a limb according to its NPP reduces muscle activity, and therefore the metabolic energy demand. In this context, good convergence between fore‐ and hindlimbs should facilitate limb movement because the freely chosen frequency for the four limbs would not (theoretically) require any muscle activity, making locomotion more efficient. When we considered the limbs in a straight position (which is not a behavioural posture in baboons but allows interspecies comparisons), we found that forelimbs and hindlimbs in females converge to the same NPP up to 3 years of age, and in males up to 1 year of age. After 1 year, male forelimb and hindlimb NPPs diverge significantly, i.e. the NPP of the hindlimb is always smaller than the NPP of the forelimb, although their relationship remains very close (Table 5). This divergence appears only after 3 years of age in females. Our values for adult male [mean (95% CI): 3.25 (1.05)] and female [2.48 (0.55)] *Papio anubis* are consistent with those found by Raichlen (2004) in adult *Papio cynocephalus* [2.35 (1.36)]. Otherwise, the values we obtained for infant baboons [i.e. between 0 and 2 years of age, females: 2.11 (0.33), males: 2.13 (0.3)] are close to those obtained by Myers & Steudel (1997) for *Canis familiaris* [1.83 (1.26)]. Consequently, very young baboons, although immature in terms of neuro‐muscular control and with distally heavy limbs, exhibit limb mechanical properties that should facilitate the onset of quadrupedal walking (see Druelle et al. in press).

The findings of this study are of great interest because this biomechanical optimization (through NPP convergence) appears to predispose infant baboons for quadrupedal walking. According to Rose (1977), the first locomotor experience of infants occurs on the ground, just a few days after birth, when they squirm and tumble forwards as the hindlimbs start making propulsive movements while the forelimbs are unable to coordinate. After a few weeks, the fore‐ and hindlimbs seem sufficiently coordinated for quadrupedal walking. We also observed that this mode dominates their repertoire from the first locomotor stages (Druelle et al. 2016b) and is very soon performed efficiently (biomechanically speaking; Raichlen, 2006). Two important developmental processes are therefore described here: in parallel with major changes in limb shape that are related to changing functional demands, baboons exhibit ‘early biomechanical optimization’ for quadrupedal locomotion that certainly favours a rapid developmental transition toward this mode. This is in line with a growing body of literature showing that infant primates have a musculoskeletal system characterized by a capacity for a particular type of performance that could compensate for the immaturity of the system and thus promote survival during the early stages of life (e.g. Ruff, 2003; Young, 2005; Young et al. 2010a; Young & Heard‐Booth, 2016). In an evolutionary perspective, early morphological adaptation of the limbs as observed in olive baboons may point to a high degree of integration between the fore‐ and hindlimbs (Young et al. 2010b). Such a developmental pattern (at the genotype level) is likely to be highly constrained by few possibilities for character variability, which would result in low degree of limb evolvability in baboons (Wagner & Altenberg, 1996).

To summarize, the changes in limb shapes and axial body related to differential growth (allometry) are significant during the ontogeny of olive baboons and appear clearly related to changes in functional demands. Besides this growth pattern, an early morphological predisposition for quadrupedal locomotion appears to be present. Similarities with the pattern of growth of Catarrhini species can be easily described, such as the proximal shift in limb mass distribution, as well as the caudal migration of the body centre of mass into the trunk; nevertheless, more ontogenetic data are necessary on Asian hominoids to confirm this Catarrhini‐related growth pattern. Furthermore, the NPP convergence observed in infant baboons does not seem to be present in other Catarrhini species, at least in chimpanzees (Schoonaert et al. 2007). Such a morphological adaptation points to a high degree of locomotor specialization for quadrupedal locomotion in baboons, which does not, however, impede their other modes of locomotion. Consequently, studying variations in the morphological development of species that are phylogenetically close appears to be important to better understand the locomotor profiles exhibited in adult extant primates and how they might have evolved.

## Author contributions

G.B. designed the study and provided the financial and material resources. G.B., F.D., K.A. and V.M. conducted the experiments and acquired the data. F.D. and G.B. analyzed the experimental data. F.D., G.B. and P.A. interpreted the results and drafted the first version of the manuscript. All the authors revised the manuscript and approved the final draft.

## Supporting information


**Table S1.** Information on the subjects.
**Table S2.** (a) Individual growth pattern of segment lengths represented by the linear regression model (*y* = a*age + *b*) in females. (b) Individual growth pattern of segment masses represented by the linear regression model (*y* = a*age + *b*) in females. (c) Individual growth pattern of segment inertia (*x* axis) represented by the linear regression model (*y* = a*age + *b*) in females. (d) Individual growth pattern of segment inertia (*y* axis) represented by the linear regression model (*y* = a*age + *b*) in females.
**Table S3.** (a) Individual growth pattern of segment lengths represented by the linear regression model (*y* = a*age + *b*) in males. (b) Individual growth pattern of segment masses represented by the linear regression model (*y* = a*age + *b*) in males. (c) Individual growth pattern of segment inertia (*x* axis) represented by the linear regression model (*y* = a*age + *b*) in males. (d) Individual growth pattern of segment inertia (*y* axis) represented by the linear regression model (*y* = a*age + *b*) in males.
**Table S4.** Individual linear regression models (RMA) in female baboons.
**Table S5.** Individual linear regression models (RMA) in male baboons.

## References

[joa12602-bib-0001] Adolph KE , Avolio AM (2000) Walking infants adapt locomotion to changing body dimensions. J Exp Psychol Human 26, 1148–1166. 10.1037//0096-1523.26.3.1148 10884014

[joa12602-bib-0003] Adolph KE , Cole WG , Komati M , et al. (2012) How do you learn to walk? Thousands of steps and dozens of falls per day. Psychol Sci 23, 1387–1394. 23085640 10.1177/0956797612446346 PMC3591461

[joa12602-bib-0004] Altmann J , Samuels A (1992) Costs of maternal care: infant‐carrying in baboons. Behav Ecol Sociobiol 29, 391–398.

[joa12602-bib-0005] Anvari Z , Berillon G , Asgari Khaneghah A , et al. (2014) Kinematics and spatiotemporal parameters of infant‐carrying in olive baboons. Am J Phys Anthropol 155, 392–404. 25059514 10.1002/ajpa.22576

[joa12602-bib-0007] Berillon G , Daver G , D'Août K , et al. (2010) Bipedal versus quadrupedal hind limb and foot kinematics in a captive sample of *Papio anubis*: setup and preliminary results. Int J Primatol 31, 159–180.

[joa12602-bib-0010] Coelho A (1985) Baboon dimorphism: growth in weight, length and adiposity from birth to 8 years of age In: Nonhuman primate models for human growth and development (ed. Watts ES), pp. 125–159. New York: John Wiley & Sons Canada.

[joa12602-bib-0011] Crompton RH , Li Y , Alexander RM , et al. (1996) Segment inertial properties of primates: new techniques for laboratory and field studies of locomotion. Am J Phys Anthropol 99, 547–570. 8779338 10.1002/(SICI)1096-8644(199604)99:4<547::AID-AJPA3>3.0.CO;2-R

[joa12602-bib-0012] Demes B , Larson S , Stern J , et al. (1994) The kinetics of primate quadrupedalism: ‘hindlimb drive’ reconsidered. J Hum Evol 26, 353–374.

[joa12602-bib-0014] Doran DM (1997) Ontogeny of locomotion in mountain gorillas and chimpanzees. J Hum Evol 32, 323–344. 9085185 10.1006/jhev.1996.0095

[joa12602-bib-0015] Druelle F , Berillon G (2013) Bipedal behaviour in olive baboons: infants versus adults in a captive environment. Folia Primatol 84, 347–361. 23969888 10.1159/000353115

[joa12602-bib-0016] Druelle F , Aerts P , Berillon G (2016a) Bipedality from locomotor autonomy to adulthood in captive olive baboon (*Papio anubis*): cross‐sectional follow‐up and first insight into the impact of body mass distribution. Am J Phys Anthropol 159, 73–84. 26293421 10.1002/ajpa.22837

[joa12602-bib-0017] Druelle F , Aerts P , Berillon G (2016b) Effect of body mass distribution on the ontogeny of positional behaviors in non‐human primates: longitudinal follow‐up of infant captive olive baboons (*Papio anubis*). Am J Primatol 78, 1201–1221. 27310368 10.1002/ajp.22575

[joa12602-bib-0018] Druelle F , Berillon G , Aerts P (In press) Intrinsic limb morpho‐dynamics and the early development of interlimb coordination of walking in a quadrupedal primate. J Zool. Doi:10.1111/jzo.12423.

[joa12602-bib-0019] Dunbar DC , Badam GL (1998) Development of posture and locomotion in free‐ranging primates. Neurosci Biobehav Rev 22, 541–546. 9595567 10.1016/s0149-7634(97)00042-0

[joa12602-bib-0020] Frontier S (1976) Etude de la décroissance des valeurs propres dans une analyse en composantes principales: comparaison avec le modèle du bâton brisé. J Exp Mar Biol Ecol 25, 67–75.

[joa12602-bib-0021] Garwicz M , Christensson M , Psouni E (2009) A unifying model for timing of walking onset in humans and other mammals. Proc Natl Acad Sci U S A 106, 21889–21893. 20018704 10.1073/pnas.0905777106 PMC2799813

[joa12602-bib-0022] Glassman DM , Coelho AM (1987) Principal components analysis of physical growth in savannah baboons. Am J Phys Anthropol 72, 59–66. 3826328 10.1002/ajpa.1330720108

[joa12602-bib-0023] Glassman D , Coelho A Jr , Carey KD , et al. (1983) Weight growth in savannah baboons: a longitudinal study from birth to adulthood. Growth 48, 425–433. 6532900

[joa12602-bib-0024] Hof AL (1996) Scaling gait data to body size. Gait Posture 4, 222–223. 10.1016/s0966-6362(01)00097-2 11323225

[joa12602-bib-0025] Hunt KD , Cant J , Gebo D , et al. (1996) Standardized descriptions of primate locomotor and postural modes. Primates 37, 363–387.

[joa12602-bib-0026] Isler K , Payne RC , Günther MM , et al. (2006) Inertial properties of hominoid limb segments. J Anat 209, 201–218. 16879599 10.1111/j.1469-7580.2006.00588.x PMC2100316

[joa12602-bib-0028] Jungers WL , Fleagle JG (1980) Postnatal growth allometry of the extremities in *Cebus albifrons* and *Cebus apella*: a longitudinal and comparative study. Am J Phys Anthropol 53, 471–478. 7468784 10.1002/ajpa.1330530403

[joa12602-bib-0029] Kilbourne BM , Hoffman LC (2013) Scale effects between body size and limb design in quadrupedal mammals. PLoS One 8, e78392. 24260117 10.1371/journal.pone.0078392 PMC3832634

[joa12602-bib-0030] Kimura T , Yaguramaki N (2009) Development of bipedal walking in humans and chimpanzees: a comparative study. Folia Primatol 80, 45–62. 19325243 10.1159/000209676

[joa12602-bib-0031] Larson SG , Schmitt D , Lemelin P , et al. (2001) Limb excursion during quadrupedal walking: how do primates compare to other mammals? J Zool 255, 353–365.

[joa12602-bib-0032] Lawler RR (2006) Sifaka positional behavior: ontogenetic and quantitative genetic approaches. Am J Phys Anthropol 131, 261–271. 16596593 10.1002/ajpa.20430

[joa12602-bib-0033] Leigh SR , VandeBerg JL , Williams‐Blangero S , et al. (2009) Growth and Development of Baboons In: The Baboon in Biomedical Research (eds Vandeberg JL, Williams‐Blangero S, Tardif S), pp. 57–88. New York: Springer.

[joa12602-bib-0034] Lemelin P , Schmitt D (2007) Origins of grasping and locomotor adaptations in primates: comparative and experimental approaches using an opossum model In: Primate origins: adaptations and evolution (eds Ravosa MJ, Dagosto M), pp. 329–380. New York: Springer.

[joa12602-bib-0035] Miller DI , Nelson RC , Goldfuss AJ (1973) Biomechanics of Sport: a Research Approach, p. 27 Philadelphia: Lea & Febiger.

[joa12602-bib-0036] Myers MJ , Steudel K (1997) Morphological conservation of limb natural pendular period in the domestic dog (*Canis familiaris*): implications for locomotor energetics. J Morphol 234, 183–196. 9360320 10.1002/(SICI)1097-4687(199711)234:2<183::AID-JMOR5>3.0.CO;2-D

[joa12602-bib-0037] Nakano Y (1996) Footfall patterns in the early development of the quadrupedal walking of Japanese macaques. Folia Primatol 66, 113–125. 8953754 10.1159/000157189

[joa12602-bib-0039] Patel BA , Horner AM , Thompson NE , et al. (2013) Ontogenetic scaling of fore‐ and hind limb posture in wild chacma baboons (*Papio hamadryas ursinus*). PLoS One 8, e71020. 23923046 10.1371/journal.pone.0071020 PMC3726614

[joa12602-bib-0040] Pouydebat E , Fragaszy D , Kivell TL (2014) Grasping in primates: for feeding, moving and human specificities. BMSAP 26, 129–133.

[joa12602-bib-0041] Preuschoft H (2004) Mechanisms for the acquisition of habitual bipedality: are there biomechanical reasons for the acquisition of upright bipedal posture? J Anat 204, 363–384. 15198701 10.1111/j.0021-8782.2004.00303.x PMC1571303

[joa12602-bib-0042] Preuschoft H , Günther M (1994) Biomechanics and body shape in primates compared with horses. Z Morphol Anthropol 80, 149–165.

[joa12602-bib-0043] Preuschoft H , Witte H , Christian A , et al. (1996) Size influences on primate locomotion and body shape, with special emphasis on the locomotion of ‘small mammals’. Folia Primatol 66, 93–112. 8953753 10.1159/000157188

[joa12602-bib-0044] Raichlen DA (2004) Convergence of forelimb and hindlimb Natural Pendular Period in baboons (*Papio cynocephalus*) and its implication for the evolution of primate quadrupedalism. J Hum Evol 46, 719–738. 15183672 10.1016/j.jhevol.2004.04.002

[joa12602-bib-0045] Raichlen DA (2005a) Effects of limb mass distribution on the ontogeny of quadrupedalism in infant baboons (*Papio cynocephalus*) and implications for the evolution of primate quadrupedalism. J Hum Evol 49, 415–431. 15998533 10.1016/j.jhevol.2005.05.004

[joa12602-bib-0046] Raichlen DA (2005b) Ontogeny of limb mass distribution in infant baboons (*Papio cynocephalus*). J Hum Evol 49, 452–467. 16011842 10.1016/j.jhevol.2005.05.005

[joa12602-bib-0047] Raichlen DA (2006) Effects of limb mass distribution on mechanical power outputs during quadrupedalism. J Exp Biol 209, 633–644. 16449558 10.1242/jeb.02061

[joa12602-bib-0048] Raichlen DA , Pontzer H , Shapiro LJ , et al. (2009) Understanding hind limb weight support in chimpanzees with implications for the evolution of primate locomotion. Am J Phys Anthropol 138, 395–402. 19003921 10.1002/ajpa.20952

[joa12602-bib-0049] Rose M (1977) Positional behaviour of olive baboons (*Papio anubis*) and its relationship to maintenance and social activities. Primates 18, 59–116.

[joa12602-bib-0050] Ruff C (2003) Ontogenetic adaptation to bipedalism: age changes in femoral to humeral length and strength proportions in humans, with a comparison to baboons. J Hum Evol 45, 317–349. 14585245 10.1016/j.jhevol.2003.08.006

[joa12602-bib-0051] Sarringhaus L , MacLatchy L , Mitani J (2014) Locomotor and postural development of wild chimpanzees. J Hum Evol 66, 29–38. 24238359 10.1016/j.jhevol.2013.09.006

[joa12602-bib-0053] Schoonaert K , D'Août K , Aerts P (2007) Morphometrics and inertial properties in the body segments of chimpanzees (*Pan troglodytes*). J Anat 210, 518–531. 17451529 10.1111/j.1469-7580.2007.00720.x PMC2375742

[joa12602-bib-0054] Shapiro L , Raichlen D (2006) Limb proportions and the ontogeny of quadrupedal walking in infant baboons (*Papio cynocephalus*). J Zool 269, 191–203.

[joa12602-bib-0057] Turnquist JE , Wells JP (1994) Ontogeny of locomotion in rhesus macaques (*Macaca mulatta*): I. Early postnatal ontogeny of the musculoskeletal system. J Hum Evol 26, 487–499.

[joa12602-bib-0058] Van Dam M , Hallemans A , Truijen S , et al. (2010) A cross‐sectional study about the relationship between morphology and step‐time parameters in children between 15 and 36 months. Gait Posture 32, 400–404. 20655227 10.1016/j.gaitpost.2010.06.020

[joa12602-bib-0059] Van Dam M , Hallemans A , Truijen S , et al. (2011) A cross‐sectional study about the relationship between morphology and kinematic parameters in children between 15 and 36 months. Gait Posture 34, 159–163. 21550246 10.1016/j.gaitpost.2011.04.001

[joa12602-bib-0060] Wagner GP , Altenberg L (1996) Perspective: complex adaptations and the evolution of evolvability. Evolution 50, 967–976. 10.1111/j.1558-5646.1996.tb02339.x 28565291

[joa12602-bib-0061] Wells JP , Turnquist JE (2001) Ontogeny of locomotion in rhesus macaques (*Macaca mulatta*): II. Postural and locomotor behavior and habitat use in a free‐ranging colony. Am J Phys Anthropol 115, 80–94. 11309753 10.1002/ajpa.1059

[joa12602-bib-0063] Wickler SJ , Hoyt DF , Clayton HM , et al. (2004) Energetic and kinematic consequences of weighting the distal limb. Equine Vet J 36, 772–777. 15656514 10.2746/0425164044848046

[joa12602-bib-0064] Witte H , Preuschoft H , Recknagel S (1991) Human body proportions explained on the basis of biomechanical principles. Z Morphol Anthropol 78, 407–423. 1887666

[joa12602-bib-0065] Young JW (2005) Ontogeny of muscle mechanical advantage in capuchin monkeys (*Cebus albifrons* and *Cebus apella*). J Zool 267, 351–362.

[joa12602-bib-0066] Young JW , Heard‐Booth AN (2016) Grasping primate development: ontogeny of intrinsic hand and foot proportions in capuchin monkeys (*Cebus albifrons* and *Sapajus apella*). Am J Phys Anthropol 161, 104–115. 27324663 10.1002/ajpa.23013

[joa12602-bib-0067] Young JW , Patel BA , Stevens NJ (2007) Body mass distribution and gait mechanics in fat‐tailed dwarf lemurs (*Cheirogaleus medius*) and patas monkeys (*Erythrocebus patas*). J Hum Evol 53, 26–40. 17512970 10.1016/j.jhevol.2007.01.005

[joa12602-bib-0068] Young JW , Fernàndez D , Fleagle JG (2010a) Ontogeny of long bone geometry in capuchin monkeys (*Cebus albifrons* and *Cebus apella*): implications for locomotor development and life history. Biol Lett 6, 197–200. 19864273 10.1098/rsbl.2009.0773 PMC2865070

[joa12602-bib-0069] Young NM , Wagner GP , Hallgrimsson B (2010b) Development and the evolvability of human limbs. Proc Natl Acad Sci USA 107, 3400–3405. 20133636 10.1073/pnas.0911856107 PMC2840520

