# Supplementary material for: Segmental morphometrics of the olive baboon (Papio anubis): a longitudinal study from birth to adulthood
Source: J Anat. 2017 Mar 14;230(6):805–19. doi: 10.1111/joa.12602 (PMC5442150; doi:10.1111/joa.12602)
Supplement: Supplementary file 1 — Table S1. Information on the subjects. Table S2. (a) Individual growth pattern of segment lengths represented by the linear regression model (y = a*age + b) in females. (b) Individual growth pattern of segment masses represented by the linear regression model (y = a*age + b) in females. (c) Individual growth pattern of segment inertia (x axis) represented by the linear regression model (y = a*age + b) in females. (d) Individual growth pattern of segment inertia (y axis) represented by the linear regression model (y = a*age + b) in females. Table S3. (a) Individual growth pattern of segment lengths represented by the linear regression model (y = a*age + b) in males. (b) Individual growth pattern of segment masses represented by the linear regression model (y = a*age + b) in males. (c) Individual growth pattern of segment inertia (x axis) represented by the linear regression model (y = a*age + b) in males. (d) Individual growth pattern of segment inertia (y axis) represented by the linear regression model (y = a*age + b) in males. Table S4. Individual linear regression models (RMA) in female baboons. Table S5. Individual linear regression models (RMA) in male baboons. [file JOA-230-805-s001.docx]

**Supplementary material**

| **TABLE S1.** *Information on the subjects* | | | | | |
| --- | --- | --- | --- | --- | --- |
| **Female (n=14)** | | | **Male (n=16)** | | |
| **Subject** | **Min-Max Age (y:year, m:month)** | **No. recorded sessions** | **Subject** | **Min-Max Age (y:year, m:month)** | **No. recorded sessions** |
| V913G | 1y7m-6y3m | 12 | V908H | 1y6m-6y1m | 12 |
| V935E | 1y8m-6y3m | 12 | V894G | 1y7m-6y3m | 12 |
| V936F | 1y4m-6y0m | 12 | V902F | 1y8m-6y3m | 12 |
| V915G | 1y4m-6y0m | 12 | V937E | 1y6m-6y1m | 12 |
| V936G | 0y2m-6y1m | 17 | V916F | 1y1m-6y3m | 15 |
| V902G | 0y4m-6y0m | 17 | V896G | 1y4m-5y11m | 13 |
| V908I | 0y5m-6y1m | 17 | V935F | 0y6m-4y8m | 13 |
| V894H | 0y6m-6y1m | 16 | V916G | 0y5m-6y1m | 17 |
| V792BB | 0y3m-7y0m | 19 | V792BA | 0y2m-6y4m | 18 |
| V916DA | 0y5m-5y2m | 17 | V894I | 0y2m-7y8m | 20 |
| V916I | 0y2m-4y0m | 14 | V896BB | 0y3m-7y10m | 20 |
| V993EB | 0y3m-6y9m | 18 | V908EA | 0y5m-7y2m | 19 |
| V936H | 0y3m-6y10m | 18 | V908J | 0y2m-7y11m | 21 |
| V908GA | 0y3m-7y0m | 19 | V935G | 0y4m-6y0m | 17 |
|  |  |  | V916H | 0y2m-5y11m | 19 |
|  |  |  | V908K | 0y3m-6y9m | 17 |

| **TABLE S2.a** Individual growth pattern of segment lengths represented by the linear regression model (y=a*age+b) in females | | | | | | | | | | | | | | | | | | | | | | | | | | | | | | | | | | | | | | |
| --- | --- | --- | --- | --- | --- | --- | --- | --- | --- | --- | --- | --- | --- | --- | --- | --- | --- | --- | --- | --- | --- | --- | --- | --- | --- | --- | --- | --- | --- | --- | --- | --- | --- | --- | --- | --- | --- | --- |
|  | Hand |  | Forearm | | | Upper arm | | | | Thigh | |  | | Shank | | |  | | Trunk | |  | | | Head | | |  | | | Tail |  | | Foot | | |  | | |
|  | a | b | a | b | | a | b | | | a | | b | | a | | | b | | a | | b | | | a | | | b | | | a | b | | a | | | b | | |
| V913G | 4.67 | 88.48 | 13.77 | 122.97 | | 16.57 | 106.18 | | | 11.12 | | 121.82 | | 12.26 | | | 120.75 | | 31.01 | | 258.58 | | | 6.94 | | | 122.91 | | | 21.67 | 302.74 | | 8.66 | | | 125.61 | | |
| V935E | 5.15 | 88.20 | 11.41 | 131.31 | | 12.08 | 121.71 | | | 9.18 | | 137.90 | | 11.13 | | | 128.95 | | 26.48 | | 258.05 | | | 9.64 | | | 120.61 | | | 16.53 | 321.71 | | 7.50 | | | 133.13 | | |
| V936F | 5.61 | 89.12 | 15.74 | 113.73 | | 14.67 | 106.97 | | | 13.25 | | 117.52 | | 12.87 | | | 120.18 | | 28.48 | | 252.86 | | | 10.04 | | | 116.76 | | | 16.53 | 307.21 | | 9.42 | | | 128.50 | | |
| V915G | 5.26 | 81.79 | 12.88 | 115.53 | | 11.86 | 112.32 | | | 11.75 | | 121.28 | | 12.19 | | | 119.26 | | 24.70 | | 251.08 | | | 8.51 | | | 117.09 | | | 12.36 | 272.43 | | 7.78 | | | 121.48 | | |
| V936G | 7.56 | 79.25 | 19.36 | 104.29 | | 17.51 | 95.09 | | | 16.12 | | 103.71 | | 16.67 | | | 104.08 | | 39.33 | | 205.38 | | | 11.27 | | | 109.12 | | | 25.12 | 258.29 | | 12.76 | | | 111.84 | | |
| V902G | 7.26 | 73.88 | 17.42 | 102.65 | | 15.48 | 96.99 | | | 17.05 | | 99.45 | | 16.72 | | | 99.40 | | 36.15 | | 196.77 | | | 10.29 | | | 109.21 | | | 18.23 | 234.55 | | 11.80 | | | 106.59 | | |
| V908I | 7.25 | 86.03 | 16.87 | 115.59 | | 14.95 | 116.39 | | | 14.96 | | 119.56 | | 15.69 | | | 120.80 | | 35.29 | | 245.41 | | | 10.10 | | | 123.09 | | | 27.14 | 325.59 | | 11.05 | | | 125.48 | | |
| V894H | 7.33 | 85.49 | 19.59 | 110.43 | | 14.91 | 113.33 | | | 18.96 | | 108.42 | | 17.78 | | | 109.82 | | 29.33 | | 248.35 | | | 12.96 | | | 109.32 | | | 25.46 | 318.23 | | 13.09 | | | 119.49 | | |
| V792BB | 7.28 | 83.20 | 15.86 | 114.84 | | 13.23 | 107.99 | | | 15.14 | | 111.27 | | 13.81 | | | 116.23 | | 30.46 | | 254.17 | | | 11.05 | | | 117.82 | | | 19.94 | 292.44 | | 10.74 | | | 122.45 | | |
| V916DA | 8.82 | 76.10 | 20.41 | 101.43 | | 19.14 | 92.94 | | | 20.64 | | 98.04 | | 20.47 | | | 101.66 | | 43.03 | | 225.47 | | | 11.68 | | | 110.80 | | | 33.01 | 263.20 | | 14.30 | | | 108.63 | | |
| V916I | 12.19 | 76.58 | 25.29 | 102.55 | | 22.79 | 93.33 | | | 22.58 | | 101.22 | | 22.24 | | | 103.50 | | 43.50 | | 222.96 | | | 14.93 | | | 112.21 | | | 43.30 | 287.04 | | 19.30 | | | 112.96 | | |
| V993EB | 8.78 | 76.18 | 17.31 | 109.63 | | 16.72 | 94.52 | | | 15.41 | | 102.79 | | 15.33 | | | 107.74 | | 34.65 | | 240.99 | | | 11.63 | | | 114.79 | | | 25.28 | 290.90 | | 12.20 | | | 115.98 | | |
| V936H | 8.14 | 81.34 | 17.69 | 111.97 | | 15.95 | 100.15 | | | 15.56 | | 103.49 | | 15.53 | | | 111.41 | | 27.46 | | 226.79 | | | 10.37 | | | 116.64 | | | 4.47 | 305.39 | | 12.19 | | | 119.10 | | |
| V908GA | 8.15 | 79.58 | 18.36 | 110.04 | | 18.28 | 97.75 | | | 17.88 | | 103.68 | | 17.31 | | | 110.19 | | 39.88 | | 221.20 | | | 11.78 | | | 111.43 | | | 24.57 | 289.53 | | 12.27 | | | 118.18 | | |
| **Mean** | **7.39** | **81.80** | **17.28** | **111.92** | | **16.01** | **103.98** | | | **15.69** | | **110.73** | | **15.71** | | | **112.43** | | **33.55** | | **236.29** | | | **10.80** | | | **115.13** | | | **22.40** | **290.66** | | **11.65** | | | **119.24** | | |
| **SD** | **1.93** | **5.10** | **3.46** | **8.27** | | **2.89** | **9.45** | | | **3.67** | | **11.42** | | **3.18** | | | **8.79** | | **6.19** | | **19.93** | | | **1.91** | | | **4.93** | | | **9.27** | **26.14** | | **3.00** | | | **7.61** | | |
| **Shapiro-Wilk (Normal distribution test)** | | | | | | | | |  | |  | |  | | |  | | |  | | |  | | |  | | |  | | |  | | | |  | |  | |
| **Obs Test Stat** | 0.91 | 0.94 | 0.96 | 0.92 | | 0.95 | 0.92 | | | 0.99 | | 0.88 | | 0.96 | | | 0.96 | | 0.94 | | 0.90 | | | 0.96 | | | 0.92 | | | 0.96 | 0.95 | | 0.91 | | | 0.99 | | |
| **P-value** | 0.14 | 0.42 | 0.71 | 0.22 | | 0.59 | 0.23 | | | 0.98 | | 0.07 | | 0.64 | | | 0.62 | | 0.45 | | 0.13 | | | 0.75 | | | 0.20 | | | 0.68 | 0.57 | | 0.16 | | | 0.99 | | |
| **Comparison with the general pattern** | | | | |  | | |  | |  | |  | | |  | | |  | |  | | |  | | |  | | |  | | |  | |  | | | |  |
| **FULL DATASET** | **7.35** | **81.19** | **17.11** | **111.14** | | **16.02** | **102.67** | | | **15.82** | | **108.61** | | **15.62** | | | **111.39** | | **33.58** | | **233.79** | | | **10.75** | | | **114.72** | | | **20.92** | **291.90** | | **11.46** | | | **118.61** | | |
| **Obs Test Stat** | 0.07 | 0.45 | 0.19 | 0.36 | | -0.01 | 0.52 | | | 0.89 | | 0.69 | | 0.11 | | | 0.44 | | -0.02 | | 0.47 | | | 0.10 | | | 0.31 | | | 0.60 | -0.18 | | 0.24 | | | 0.31 | | |
| **P-value** | 0.94 | 0.66 | 0.85 | 0.73 | | 0.99 | 0.61 | | | -0.14 | | 0.50 | | 0.91 | | | 0.67 | | 0.99 | | 0.65 | | | 0.92 | | | 0.76 | | | 0.56 | 0.86 | | 0.81 | | | 0.76 | | |

| **TABLE S2.b** Individual growth pattern of segment masses represented by the linear regression model (y=a*age+b) in females | | | | | | | | | | | | | | | | | | |
| --- | --- | --- | --- | --- | --- | --- | --- | --- | --- | --- | --- | --- | --- | --- | --- | --- | --- | --- |
|  | Hand |  | Forearm | | Upper arm | | Thigh |  | Shank |  | Trunk |  | Head |  | Tail |  | Foot |  |
|  | a | b | a | b | a | b | a | b | a | b | a | b | a | b | a | b | a | b |
| V913G | 0.01 | 0.03 | 0.04 | 0.05 | 0.08 | 0.01 | 0.12 | 0.09 | 0.04 | 0.03 | 0.98 | 0.48 | 0.14 | 0.33 | 0.03 | 0.04 | 0.03 | 0.04 |
| V935E | 0.01 | 0.04 | 0.03 | 0.07 | 0.06 | 0.05 | 0.14 | 0.12 | 0.04 | 0.05 | 1.07 | 0.93 | 0.15 | 0.38 | 0.03 | 0.05 | 0.02 | 0.07 |
| V936F | 0.01 | 0.03 | 0.04 | 0.03 | 0.07 | -0.01 | 0.14 | 0.08 | 0.04 | 0.05 | 1.00 | 0.50 | 0.18 | 0.27 | 0.03 | 0.06 | 0.03 | 0.03 |
| V915G | 0.01 | 0.03 | 0.03 | 0.05 | 0.06 | 0.03 | 0.14 | 0.05 | 0.04 | 0.04 | 0.90 | 0.33 | 0.14 | 0.27 | 0.02 | 0.06 | 0.02 | 0.04 |
| V936G | 0.01 | 0.02 | 0.04 | 0.03 | 0.07 | 0.00 | 0.14 | 0.04 | 0.05 | 0.02 | 1.04 | 0.30 | 0.16 | 0.27 | 0.03 | 0.03 | 0.03 | 0.04 |
| V902G | 0.01 | 0.02 | 0.04 | 0.02 | 0.06 | 0.02 | 0.13 | 0.02 | 0.05 | 0.01 | 0.85 | 0.13 | 0.15 | 0.27 | 0.02 | 0.03 | 0.03 | 0.03 |
| V908I | 0.02 | 0.03 | 0.04 | 0.05 | 0.08 | 0.06 | 0.21 | 0.06 | 0.06 | 0.04 | 1.26 | 0.59 | 0.19 | 0.32 | 0.05 | 0.06 | 0.04 | 0.06 |
| V894H | 0.02 | 0.02 | 0.05 | 0.04 | 0.08 | 0.03 | 0.21 | -0.03 | 0.06 | 0.01 | 1.07 | 0.53 | 0.20 | 0.31 | 0.04 | 0.06 | 0.03 | 0.05 |
| V792BB | 0.02 | 0.03 | 0.05 | 0.04 | 0.09 | 0.01 | 0.18 | 0.07 | 0.05 | 0.04 | 1.15 | 0.74 | 0.19 | 0.33 | 0.03 | 0.07 | 0.03 | 0.05 |
| V916DA | 0.02 | 0.02 | 0.06 | 0.01 | 0.07 | 0.01 | 0.18 | 0.01 | 0.05 | 0.02 | 1.24 | 0.34 | 0.18 | 0.27 | 0.04 | 0.04 | 0.03 | 0.04 |
| V916I | 0.02 | 0.02 | 0.06 | 0.02 | 0.10 | 0.00 | 0.25 | -0.03 | 0.07 | 0.02 | 1.27 | 0.37 | 0.23 | 0.25 | 0.05 | 0.04 | 0.05 | 0.03 |
| V993EB | 0.02 | 0.02 | 0.05 | 0.03 | 0.08 | 0.02 | 0.16 | 0.07 | 0.05 | 0.04 | 1.24 | 0.45 | 0.21 | 0.29 | 0.04 | 0.05 | 0.03 | 0.05 |
| V936H | 0.02 | 0.03 | 0.04 | 0.04 | 0.08 | 0.01 | 0.16 | 0.04 | 0.05 | 0.04 | 0.94 | 0.57 | 0.17 | 0.30 | 0.03 | 0.06 | 0.03 | 0.05 |
| V908GA | 0.02 | 0.02 | 0.05 | 0.03 | 0.10 | -0.02 | 0.19 | 0.01 | 0.05 | 0.03 | 1.38 | 0.19 | 0.20 | 0.26 | 0.03 | 0.05 | 0.03 | 0.05 |
| **Mean** | **0.02** | **0.03** | **0.04** | **0.04** | **0.08** | **0.02** | **0.17** | **0.04** | **0.05** | **0.03** | **1.10** | **0.46** | **0.18** | **0.29** | **0.03** | **0.05** | **0.03** | **0.05** |
| **SD** | **0.00** | **0.01** | **0.01** | **0.02** | **0.01** | **0.02** | **0.04** | **0.04** | **0.01** | **0.01** | **0.16** | **0.21** | **0.03** | **0.04** | **0.01** | **0.01** | **0.01** | **0.01** |
| **Shapiro-Wilk (Normal distribution test)** | | | | | |  |  |  |  |  |  |  |  |  |  |  |  |  |
| **Obs Test Stat** | 0.94 | 0.95 | 0.93 | 0.98 | 0.94 | 0.96 | 0.94 | 0.97 | 0.95 | 0.95 | 0.96 | 0.97 | 0.95 | 0.92 | 0.97 | 0.97 | 0.76 | 0.95 |
| **P-value** | 0.39 | 0.58 | 0.32 | 0.91 | 0.46 | 0.68 | 0.45 | 0.78 | 0.50 | 0.54 | 0.71 | 0.84 | 0.50 | 0.20 | 0.84 | 0.86 | 0.00 | 0.46 |
| **Comparison with the general pattern** | | | | |  |  |  |  |  |  |  |  |  |  |  |  |  |  |
| **FULL DATASET** | **0.02** | **0.03** | **0.04** | **0.04** | **0.08** | **0.01** | **0.17** | **0.05** | **0.05** | **0.03** | **1.10** | **0.46** | **0.18** | **0.29** | **0.03** | **0.05** | **0.03** | **0.05** |
| **Obs Test Stat** | 0.26 | -0.05 | 0.18 | 0.16 | 0.06 | 0.06 | 0.31 | -0.24 | 0.20 | 0.07 | 0.01 | 0.10 | 0.11 | 0.01 | 0.45 | -0.75 | N/A | -1.08 |
| **P-value** | 0.80 | 0.96 | 0.86 | 0.87 | 0.95 | 0.95 | 0.77 | 0.82 | 0.85 | 0.94 | 0.99 | 0.92 | 0.91 | 0.99 | 0.66 | 0.47 | N/A | 0.30 |

| **TABLE S2.c** Individual growth pattern of segment inertia (x axis) represented by the linear regression model (y=a*age+b) in females | | | | | | | | | | | | | | | | | | |
| --- | --- | --- | --- | --- | --- | --- | --- | --- | --- | --- | --- | --- | --- | --- | --- | --- | --- | --- |
|  | Hand |  | Forearm | | Upper arm | | Thigh |  | Shank |  | Trunk |  | Head |  | Tail |  | Foot |  |
|  | a | b | a | b | a | b | a | b | a | b | a | b | a | b | a | b | a | b |
| V913G | 68.54 | 7.33 | 530.31 | -361.87 | 995.19 | -1234.26 | 1139.86 | -687.89 | 551.89 | -430.71 | 79415.18 | -74746.62 | 1371.00 | 278.65 | 1157.12 | -410.96 | 203.54 | -63.95 |
| V935E | 60.72 | 31.72 | 445.46 | -38.76 | 821.01 | -702.68 | 1557.63 | -737.61 | 569.91 | -201.83 | 78633.40 | -54881.08 | 1675.09 | 226.88 | 972.77 | 261.02 | 186.96 | 77.74 |
| V936F | 69.66 | 2.59 | 525.56 | -490.73 | 781.66 | -885.36 | 1654.90 | -1387.88 | 575.18 | -298.72 | 71144.64 | -56757.96 | 1952.13 | -696.86 | 784.02 | 437.57 | 265.56 | -138.05 |
| V915G | 45.89 | 22.18 | 383.27 | -163.27 | 586.11 | -347.58 | 1407.39 | -1040.63 | 544.91 | -310.32 | 56735.08 | -47172.26 | 1393.10 | -86.57 | 373.09 | 529.64 | 159.93 | -5.49 |
| V936G | 60.67 | 10.07 | 435.49 | -160.27 | 767.74 | -551.63 | 1346.82 | -686.76 | 545.76 | -232.34 | 64816.56 | -37803.35 | 1534.71 | 88.31 | 794.80 | -10.16 | 247.98 | -40.25 |
| V902G | 53.44 | -5.06 | 425.13 | -250.49 | 547.35 | -390.73 | 1325.58 | -1075.63 | 551.06 | -419.75 | 47443.51 | -37895.74 | 1386.03 | 115.79 | 390.73 | 142.37 | 163.38 | -6.40 |
| V908I | 86.18 | 12.81 | 542.27 | -131.17 | 909.97 | -299.86 | 2232.80 | -1246.81 | 819.21 | -254.28 | 93963.02 | -58083.69 | 2035.04 | 239.60 | 1871.12 | 142.99 | 296.74 | 2.54 |
| V894H | 89.02 | -1.51 | 683.43 | -415.68 | 869.45 | -504.06 | 2522.84 | -2466.20 | 758.06 | -531.33 | 68430.55 | -30544.78 | 2185.03 | -275.66 | 1409.68 | 306.89 | 239.31 | 6.21 |
| V792BB | 68.91 | 28.87 | 627.41 | -276.79 | 862.64 | -530.54 | 1941.80 | -1215.59 | 689.57 | -277.91 | 78913.86 | -26302.40 | 2069.92 | 138.55 | 832.74 | 809.05 | 222.24 | 84.37 |
| V916DA | 77.21 | -8.95 | 610.32 | -426.78 | 809.56 | -634.84 | 1831.19 | -1425.66 | 666.51 | -365.67 | 82811.31 | -49220.09 | 1691.03 | 84.84 | 1181.65 | -151.87 | 206.38 | -3.50 |
| V916I | 107.12 | -16.05 | 691.50 | -296.34 | 991.96 | -608.12 | 2332.24 | -1481.37 | 766.64 | -332.56 | 65616.89 | -19025.73 | 2138.46 | 11.37 | 1698.54 | -80.95 | 391.43 | -75.85 |
| V993EB | 95.85 | -18.82 | 647.65 | -381.34 | 835.02 | -554.69 | 1595.49 | -882.45 | 604.62 | -189.85 | 89397.13 | -53444.12 | 2215.18 | -130.23 | 1295.43 | 26.57 | 246.28 | 58.75 |
| V936H | 76.81 | 15.35 | 530.23 | -93.51 | 838.00 | -609.26 | 1680.62 | -1060.19 | 667.92 | -288.83 | 52985.62 | -23432.73 | 1789.83 | 210.04 | 452.95 | 873.82 | 246.47 | 37.20 |
| V908GA | 84.69 | -7.17 | 696.87 | -404.80 | 1104.04 | -916.16 | 2104.19 | -1533.25 | 763.32 | -394.41 | 104708.38 | -81838.68 | 2168.89 | -428.72 | 1034.01 | 314.97 | 216.99 | 42.77 |
| **Mean** | **74.62** | **5.24** | **555.35** | **-277.99** | **837.12** | **-626.41** | **1762.38** | **-1209.14** | **648.18** | **-323.47** | **73929.65** | **-46510.66** | **1828.96** | **-16.00** | **1017.76** | **227.93** | **235.23** | **-1.71** |
| **SD** | **16.91** | **15.88** | **106.35** | **141.72** | **147.57** | **248.35** | **415.39** | **462.58** | **97.77** | **96.35** | **16100.21** | **18629.02** | **319.82** | **283.47** | **458.87** | **357.81** | **58.86** | **62.29** |
| **Shapiro-Wilk (Normal distribution test)** | | | |  |  |  |  |  |  |  |  |  |  |  |  |  |  |  |
| **Obs Test Stat** | 0.99 | 0.97 | 0.93 | 0.95 | 0.94 | 0.91 | 0.96 | 0.87 | 0.88 | 0.96 | 0.99 | 0.96 | 0.89 | 0.87 | 0.97 | 0.98 | 0.89 | 0.95 |
| **P-value** | 0.99 | 0.83 | 0.30 | 0.54 | 0.43 | 0.16 | 0.75 | 0.04 | 0.06 | 0.75 | 0.98 | 0.72 | 0.08 | 0.05 | 0.71 | 0.92 | 0.09 | 0.59 |
| **Comparison with the general pattern** | | | | |  |  |  |  |  |  |  |  |  |  |  |  |  |  |
| **FULL DATASET** | **73.45** | **6.74** | **551.64** | **-266.72** | **829.34** | **-588.09** | **1734.90** | **-1155.79** | **641.62** | **-301.36** | **74465.39** | **-45258.43** | **1830.37** | **5.08** | **957.71** | **365.70** | **222.34** | **29.11** |
| **Obs Test Stat** | 0.26 | -0.35 | 0.13 | -0.30 | 0.20 | -0.58 | 0.25 | N/A | 0.25 | -0.86 | -0.12 | -0.25 | -0.02 | N/A | 0.49 | -1.44 | 0.82 | -1.85 |
| **P-value** | 0.80 | 0.73 | 0.90 | 0.77 | 0.85 | 0.57 | 0.81 | N/A | 0.81 | 0.41 | 0.90 | 0.81 | 0.99 | N/A | 0.63 | 0.17 | 0.43 | 0.09 |

| **TABLE S2.d** Individual growth pattern of segment inertia (y axis) represented by the linear regression model (y=a*age+b) in females | | | | | | | | | | | | | | | | | | |
| --- | --- | --- | --- | --- | --- | --- | --- | --- | --- | --- | --- | --- | --- | --- | --- | --- | --- | --- |
|  | Hand |  | Forearm | | Upper arm | | Thigh |  | Shank |  | Trunk |  | Head |  | Tail |  | Foot |  |
|  | a | b | a | b | a | b | a | b | a | b | a | b | a | b | a | b | a | b |
| V913G | 65.40 | 8.86 | 528.76 | -358.81 | 1008.07 | -1246.02 | 1183.30 | -726.49 | 554.53 | -431.78 | 79303.43 | -74605.10 | 1381.27 | 277.68 | 1157.12 | -410.96 | 198.85 | -62.35 |
| V935E | 58.40 | 31.04 | 444.27 | -37.06 | 831.29 | -705.72 | 1610.61 | -777.57 | 574.17 | -205.61 | 78134.82 | -54144.33 | 1697.71 | 229.71 | 972.77 | 261.02 | 183.33 | 72.76 |
| V936F | 66.55 | 4.53 | 525.18 | -490.33 | 792.60 | -899.39 | 1686.01 | -1395.65 | 578.15 | -300.37 | 70583.90 | -56039.35 | 1968.83 | -702.26 | 784.02 | 437.57 | 260.32 | -136.52 |
| V915G | 43.85 | 22.44 | 381.96 | -162.05 | 593.87 | -353.44 | 1447.40 | -1081.54 | 548.07 | -313.86 | 56318.29 | -46452.82 | 1399.86 | -81.06 | 373.09 | 529.64 | 155.40 | -4.65 |
| V936G | 58.51 | 9.89 | 435.02 | -160.05 | 781.43 | -565.67 | 1384.96 | -710.29 | 549.10 | -234.91 | 64421.83 | -37442.93 | 1553.45 | 75.77 | 794.80 | -10.16 | 243.57 | -40.82 |
| V902G | 51.55 | -5.24 | 424.79 | -250.69 | 554.92 | -397.01 | 1358.77 | -1100.96 | 553.93 | -423.00 | 47349.93 | -37780.98 | 1387.65 | 130.95 | 390.73 | 142.37 | 159.47 | -5.95 |
| V908I | 82.45 | 12.51 | 541.83 | -131.65 | 924.67 | -306.94 | 2337.45 | -1333.92 | 825.74 | -259.12 | 93228.59 | -57305.01 | 2054.76 | 231.78 | 1871.12 | 142.99 | 290.29 | -0.14 |
| V894H | 85.86 | -0.92 | 682.04 | -414.90 | 882.81 | -513.77 | 2616.13 | -2568.50 | 763.02 | -536.67 | 68155.85 | -30162.38 | 2207.84 | -303.61 | 1409.68 | 306.89 | 232.97 | 7.16 |
| V792BB | 66.21 | 28.08 | 627.09 | -277.29 | 882.25 | -548.82 | 2007.17 | -1256.59 | 695.21 | -283.46 | 78612.86 | -26231.00 | 2099.04 | 116.13 | 832.74 | 809.05 | 216.71 | 82.87 |
| V916DA | 74.07 | -8.64 | 608.97 | -425.84 | 820.18 | -643.98 | 1885.58 | -1466.60 | 670.46 | -368.62 | 82470.19 | -48951.44 | 1714.86 | 67.48 | 1181.65 | -151.87 | 200.15 | -3.07 |
| V916I | 103.52 | -15.98 | 690.62 | -296.28 | 1007.42 | -619.41 | 2398.07 | -1520.15 | 770.54 | -334.72 | 65168.91 | -18634.35 | 2169.37 | -9.04 | 1698.54 | -80.95 | 381.30 | -73.12 |
| V993EB | 91.34 | -17.55 | 647.23 | -382.41 | 849.23 | -567.38 | 1656.01 | -924.96 | 609.31 | -194.88 | 88914.61 | -52981.84 | 2246.14 | -166.98 | 1295.43 | 26.57 | 240.31 | 57.42 |
| V936H | 74.49 | 14.06 | 529.21 | -93.48 | 853.05 | -625.16 | 1734.75 | -1100.87 | 672.39 | -291.77 | 52644.92 | -23155.14 | 1806.80 | 186.07 | 452.95 | 873.82 | 241.21 | 35.95 |
| V908GA | 81.42 | -6.84 | 695.56 | -404.13 | 1122.80 | -935.05 | 2175.69 | -1589.57 | 767.45 | -397.95 | 103944.91 | -81234.68 | 2188.27 | -438.51 | 1034.01 | 314.97 | 211.62 | 41.75 |
| **Mean** | **71.69** | **5.45** | **554.47** | **-277.50** | **850.33** | **-637.70** | **1820.14** | **-1253.83** | **652.29** | **-326.91** | **73518.07** | **-46080.10** | **1848.28** | **-27.56** | **1017.76** | **227.93** | **229.68** | **-2.05** |
| **SD** | **16.29** | **15.45** | **106.29** | **141.69** | **150.33** | **250.17** | **433.57** | **477.88** | **98.59** | **96.19** | **15976.76** | **18533.58** | **326.62** | **285.64** | **458.87** | **357.81** | **57.49** | **60.82** |
| **Shapiro-Wilk (Normal distribution test)** | | | |  |  |  |  |  |  |  |  |  |  |  |  |  |  |  |
| **Obs Test Stat** | 0.99 | 0.96 | 0.93 | 0.95 | 0.94 | 0.91 | 0.96 | 0.86 | 0.88 | 0.96 | 0.98 | 0.96 | 0.89 | 0.88 | 0.96 | 0.98 | 0.90 | 0.95 |
| **P-value** | 0.99 | 0.74 | 0.30 | 0.55 | 0.43 | 0.16 | 0.68 | 0.03 | 0.06 | 0.69 | 0.98 | 0.71 | 0.09 | 0.06 | 0.71 | 0.92 | 0.10 | 0.58 |
| **Comparison with the general pattern** | | | | | |  |  |  |  |  |  |  |  |  |  |  |  |  |
| **FULL DATASET** | **70.58** | **6.71** | **550.84** | **-266.53** | **843.05** | **-600.66** | **1793.15** | **-1200.92** | **645.83** | **-305.03** | **74056.72** | **-44859.45** | **1849.83** | **-8.40** | **957.71** | **365.70** | **217.17** | **27.98** |
| **Obs Test Stat** | 0.25 | -0.31 | 0.13 | -0.29 | 0.18 | -0.55 | 0.23 | N/A | 0.25 | -0.85 | -0.13 | -0.25 | -0.02 | -0.25 | 0.49 | -1.44 | 0.81 | -1.85 |
| **P-value** | 0.80 | 0.76 | 0.90 | 0.78 | 0.86 | 0.59 | 0.82 | N/A | 0.81 | 0.41 | 0.90 | 0.81 | 0.99 | 0.81 | 0.63 | 0.17 | 0.43 | 0.09 |

| **TABLE S3.a** Individual growth pattern of segment lengths represented by the linear regression model (y=a*age+b) in males | | | | | | | | | | | | | | | | | | |
| --- | --- | --- | --- | --- | --- | --- | --- | --- | --- | --- | --- | --- | --- | --- | --- | --- | --- | --- |
|  | Hand |  | Forearm | | Upper arm | | Thigh |  | Shank |  | Trunk |  | Head |  | Tail |  | Foot |  |
|  | a | b | a | b | a | b | a | b | a | b | a | b | a | b | a | b | a | b |
| V908H | 8.21 | 83.04 | 20.27 | 103.36 | 19.65 | 98.92 | 17.84 | 104.90 | 19.45 | 104.29 | 39.54 | 246.81 | 16.69 | 103.83 | 35.09 | 275.82 | 15.35 | 110.98 |
| V894G | 10.40 | 83.51 | 20.98 | 114.11 | 19.21 | 104.84 | 20.51 | 107.76 | 21.86 | 103.48 | 45.44 | 207.18 | 18.05 | 109.59 | 40.16 | 284.48 | 16.32 | 118.01 |
| V902F | 7.30 | 84.14 | 18.08 | 113.27 | 19.75 | 104.39 | 17.21 | 112.37 | 17.97 | 108.54 | 41.83 | 213.05 | 17.41 | 101.60 | 23.26 | 194.01 | 14.02 | 113.95 |
| V937E | 8.02 | 87.58 | 21.64 | 106.35 | 19.19 | 102.76 | 16.74 | 117.49 | 18.39 | 110.56 | 40.66 | 239.61 | 16.34 | 108.03 | 34.63 | 270.03 | 15.02 | 118.45 |
| V916F | 8.31 | 87.34 | 18.03 | 111.01 | 16.47 | 111.39 | 16.12 | 120.21 | 17.96 | 110.47 | 37.81 | 253.06 | 16.79 | 106.74 | 31.20 | 289.01 | 13.52 | 120.37 |
| V896G | 10.32 | 86.35 | 20.77 | 112.21 | 21.37 | 98.03 | 22.17 | 100.79 | 22.17 | 105.93 | 45.00 | 229.35 | 18.70 | 105.34 | 39.06 | 297.57 | 15.36 | 124.71 |
| V935F | 10.02 | 78.33 | 24.27 | 101.13 | 20.76 | 107.54 | 20.47 | 102.90 | 20.76 | 105.54 | 39.36 | 240.17 | 14.13 | 113.30 | 36.85 | 270.88 | 15.87 | 114.47 |
| V916G | 11.72 | 79.38 | 21.48 | 109.88 | 22.43 | 97.62 | 21.42 | 103.24 | 23.03 | 102.43 | 45.69 | 223.44 | 18.68 | 110.24 | 40.35 | 300.94 | 16.57 | 117.35 |
| V792BA | 10.89 | 80.31 | 21.37 | 111.06 | 22.28 | 91.76 | 20.31 | 104.64 | 21.66 | 106.88 | 48.38 | 216.74 | 16.77 | 111.20 | 24.57 | 285.87 | 16.76 | 114.39 |
| V894I | 9.87 | 79.08 | 20.79 | 101.96 | 20.27 | 95.52 | 19.81 | 97.24 | 19.10 | 101.96 | 40.58 | 235.15 | 16.97 | 114.18 | 35.50 | 269.49 | 15.67 | 116.53 |
| V896BB | 9.06 | 76.29 | 20.66 | 102.57 | 19.04 | 92.61 | 18.43 | 100.20 | 19.25 | 101.49 | 37.32 | 217.18 | 15.91 | 107.23 | 32.08 | 262.68 | 14.59 | 108.16 |
| V908EA | 11.05 | 77.39 | 21.93 | 108.19 | 21.43 | 93.73 | 19.44 | 103.00 | 20.96 | 105.68 | 43.50 | 232.52 | 16.21 | 106.78 | 35.46 | 275.79 | 15.89 | 114.58 |
| V908J | 10.38 | 85.32 | 19.77 | 113.99 | 20.84 | 99.59 | 20.24 | 103.90 | 21.12 | 110.54 | 37.43 | 233.79 | 16.45 | 115.18 | 36.14 | 293.44 | 15.42 | 122.76 |
| V935G | 10.56 | 77.71 | 22.61 | 97.30 | 22.96 | 86.00 | 19.46 | 95.44 | 21.58 | 99.04 | 44.05 | 228.48 | 18.75 | 108.24 | 37.04 | 272.37 | 16.97 | 111.05 |
| V916H | 12.39 | 80.26 | 24.03 | 101.48 | 22.63 | 96.31 | 22.46 | 98.88 | 23.43 | 100.18 | 48.90 | 226.28 | 18.67 | 110.47 | 43.07 | 279.07 | 17.79 | 113.32 |
| V908K | 12.64 | 77.65 | 23.53 | 97.56 | 24.53 | 87.92 | 24.71 | 90.80 | 24.19 | 95.90 | 51.83 | 214.39 | 18.72 | 102.71 | 39.03 | 263.90 | 17.59 | 110.65 |
| **Mean** | **10.07** | **81.48** | **21.26** | **106.59** | **20.80** | **98.06** | **19.83** | **103.98** | **20.81** | **104.56** | **42.96** | **228.58** | **17.20** | **108.42** | **35.22** | **274.08** | **15.79** | **115.61** |
| **SD** | **1.56** | **3.82** | **1.81** | **5.84** | **1.95** | **6.95** | **2.26** | **7.65** | **1.95** | **4.31** | **4.39** | **12.85** | **1.32** | **3.97** | **5.36** | **24.28** | **1.19** | **4.54** |
| **Shapiro-Wilk (Normal distribution test)** | | | | |  |  |  |  |  |  |  |  |  |  |  |  |  |  |
| **Obs Test Stat** | 0.96 | 0.92 | 0.95 | 0.92 | 0.98 | 0.99 | 0.98 | 0.94 | 0.95 | 0.96 | 0.95 | 0.98 | 0.90 | 0.98 | 0.90 | 0.74 | 0.98 | 0.97 |
| **P-value** | 0.68 | 0.15 | 0.52 | 0.15 | 0.91 | 0.99 | 0.89 | 0.40 | 0.55 | 0.70 | 0.47 | 0.95 | 0.08 | 0.93 | 0.09 | 0.00 | 0.96 | 0.83 |
| **Comparison with the general pattern** | | | | | | | | | | | |  |  |  |  |  |  |  |
| **FULL DATASET** | **10.25** | **80.68** | **21.24** | **106.16** | **20.92** | **96.94** | **20.15** | **102.29** | **20.86** | **104.18** | **42.38** | **229.80** | **16.98** | **109.49** | **34.13** | **278.38** | **15.79** | **115.45** |
| **Obs Test Stat** | -0.46 | 0.84 | 0.05 | 0.29 | -0.25 | 0.64 | -0.56 | 0.89 | -0.11 | 0.35 | 0.53 | -0.38 | 0.68 | -1.08 | 0.81 | -0.71 | 0.01 | 0.14 |
| **P-value** | 0.65 | 0.42 | 0.96 | 0.77 | 0.81 | 0.53 | 0.58 | 0.39 | 0.91 | 0.73 | 0.61 | 0.71 | 0.51 | 0.30 | 0.43 | 0.49 | 0.99 | 0.89 |

| **TABLE S3.b** Individual growth pattern of segment masses represented by the linear regression model (y=a*age+b) in males | | | | | | | | | | | | | | | | | | |
| --- | --- | --- | --- | --- | --- | --- | --- | --- | --- | --- | --- | --- | --- | --- | --- | --- | --- | --- |
|  | Hand |  | Forearm | | Upper arm | | Thigh |  | Shank |  | Trunk |  | Head |  | Tail |  | Foot |  |
|  | a | b | a | b | a | b | a | b | a | b | a | b | a | b | a | b | a | b |
| V908H | 0.04 | -0.01 | 0.08 | -0.03 | 0.14 | -0.12 | 0.26 | -0.19 | 0.08 | -0.04 | 2.00 | -1.63 | 0.33 | -0.11 | 0.08 | -0.04 | 0.06 | 0.00 |
| V894G | 0.04 | 0.00 | 0.10 | -0.06 | 0.14 | -0.12 | 0.33 | -0.28 | 0.09 | -0.05 | 2.26 | -2.49 | 0.45 | -0.26 | 0.08 | -0.04 | 0.07 | -0.02 |
| V902F | 0.03 | 0.01 | 0.08 | -0.08 | 0.13 | -0.11 | 0.28 | -0.27 | 0.08 | -0.06 | 2.11 | -2.39 | 0.35 | -0.16 | 0.05 | -0.02 | 0.06 | -0.01 |
| V937E | 0.03 | 0.01 | 0.09 | -0.05 | 0.17 | -0.18 | 0.32 | -0.31 | 0.08 | -0.04 | 2.10 | -1.74 | 0.40 | -0.18 | 0.07 | -0.03 | 0.06 | 0.01 |
| V916F | 0.03 | 0.02 | 0.07 | 0.00 | 0.11 | -0.03 | 0.33 | -0.27 | 0.08 | -0.02 | 1.87 | -1.03 | 0.38 | 0.00 | 0.06 | 0.00 | 0.05 | 0.03 |
| V896G | 0.03 | 0.02 | 0.08 | -0.03 | 0.16 | -0.13 | 0.36 | -0.32 | 0.10 | -0.05 | 2.26 | -1.98 | 0.37 | -0.06 | 0.07 | -0.01 | 0.06 | 0.02 |
| V935F | 0.03 | 0.02 | 0.06 | 0.01 | 0.10 | 0.01 | 0.21 | -0.04 | 0.06 | 0.02 | 1.43 | 0.19 | 0.25 | 0.24 | 0.05 | 0.02 | 0.04 | 0.02 |
| V916G | 0.04 | 0.01 | 0.08 | -0.01 | 0.17 | -0.10 | 0.34 | -0.19 | 0.10 | -0.03 | 2.06 | -0.84 | 0.40 | 0.03 | 0.08 | 0.01 | 0.06 | 0.02 |
| V792BA | 0.03 | 0.01 | 0.07 | 0.01 | 0.14 | -0.07 | 0.28 | -0.08 | 0.08 | 0.00 | 1.95 | -0.76 | 0.38 | 0.05 | 0.05 | 0.02 | 0.05 | 0.03 |
| V894I | 0.03 | 0.01 | 0.09 | -0.03 | 0.14 | -0.05 | 0.28 | -0.07 | 0.09 | -0.01 | 1.77 | -0.37 | 0.40 | 0.04 | 0.07 | 0.02 | 0.06 | 0.03 |
| V896BB | 0.03 | 0.02 | 0.08 | -0.02 | 0.13 | -0.06 | 0.25 | -0.08 | 0.08 | -0.03 | 1.66 | -0.61 | 0.34 | 0.07 | 0.07 | 0.00 | 0.04 | 0.03 |
| V908EA | 0.03 | 0.01 | 0.09 | -0.03 | 0.15 | -0.11 | 0.36 | -0.24 | 0.10 | -0.03 | 1.90 | -0.84 | 0.36 | -0.02 | 0.06 | 0.01 | 0.05 | 0.02 |
| V908J | 0.03 | 0.03 | 0.08 | 0.02 | 0.14 | -0.03 | 0.34 | -0.11 | 0.10 | 0.00 | 1.88 | -0.20 | 0.38 | 0.10 | 0.06 | 0.03 | 0.06 | 0.05 |
| V935G | 0.03 | 0.01 | 0.08 | -0.03 | 0.15 | -0.09 | 0.31 | -0.18 | 0.08 | -0.01 | 2.07 | -1.04 | 0.36 | 0.07 | 0.07 | 0.00 | 0.06 | 0.01 |
| V916H | 0.04 | 0.01 | 0.09 | -0.01 | 0.14 | -0.04 | 0.32 | -0.14 | 0.10 | -0.03 | 2.13 | -0.78 | 0.39 | 0.12 | 0.07 | 0.01 | 0.06 | 0.03 |
| V908K | 0.04 | 0.01 | 0.08 | -0.02 | 0.16 | -0.09 | 0.30 | -0.11 | 0.10 | -0.04 | 2.03 | -0.86 | 0.37 | 0.04 | 0.07 | -0.01 | 0.05 | 0.03 |
| **Mean** | **0.03** | **0.01** | **0.08** | **-0.02** | **0.14** | **-0.08** | **0.30** | **-0.18** | **0.09** | **-0.03** | **1.97** | **-1.08** | **0.37** | **0.00** | **0.06** | **0.00** | **0.05** | **0.02** |
| **SD** | **0.00** | **0.01** | **0.01** | **0.03** | **0.02** | **0.05** | **0.04** | **0.09** | **0.01** | **0.02** | **0.22** | **0.76** | **0.04** | **0.13** | **0.01** | **0.02** | **0.01** | **0.02** |
| **Shapiro-Wilk (Normal distribution test)** | | | |  |  |  |  |  |  |  |  |  |  |  |  |  |  |  |
| **Obs Test Stat** | 0.93 | 0.96 | 0.95 | 0.97 | 0.96 | 0.97 | 0.96 | 0.92 | 0.93 | 0.97 | 0.93 | 0.94 | 0.89 | 0.96 | 0.94 | 0.95 | 0.94 | 0.90 |
| **P-value** | 0.25 | 0.64 | 0.47 | 0.75 | 0.71 | 0.88 | 0.56 | 0.20 | 0.21 | 0.75 | 0.27 | 0.35 | 0.06 | 0.72 | 0.40 | 0.42 | 0.37 | 0.07 |
| **Comparison with the general pattern** | | | | | |  |  |  |  |  |  |  |  |  |  |  |  |  |
| **FULL DATASET** | **0.03** | **0.01** | **0.08** | **-0.02** | **0.14** | **-0.07** | **0.30** | **-0.16** | **0.09** | **-0.02** | **1.91** | **-0.81** | **0.37** | **0.03** | **0.06** | **0.01** | **0.05** | **0.02** |
| **Obs Test Stat** | 1.20 | 0.70 | 0.84 | -0.31 | 0.10 | -1.12 | 0.46 | -0.85 | -0.98 | -1.17 | 1.07 | -1.44 | -0.03 | -1.05 | 2.07 | -2.14 | 2.57 | -0.43 |
| **P-value** | 0.25 | 0.50 | 0.41 | 0.76 | 0.92 | 0.28 | 0.65 | 0.41 | 0.34 | 0.26 | 0.30 | 0.17 | 0.98 | 0.31 | 0.06 | 0.05 | 0.02 | 0.67 |

| **TABLE S3.c** Individual growth pattern of segment inertia (x axis) represented by the linear regression model (y=a*age+b) in males | | | | | | | | | | | | | | | | | | |
| --- | --- | --- | --- | --- | --- | --- | --- | --- | --- | --- | --- | --- | --- | --- | --- | --- | --- | --- |
|  | Hand |  | Forearm | | Upper arm | | Thigh |  | Shank |  | Trunk |  | Head |  | Tail |  | Foot |  |
|  | a | b | a | b | a | b | a | b | a | b | a | b | a | b | a | b | a | b |
| V908H | 181.25 | -220.59 | 1241.06 | -1950.94 | 1962.82 | -3370.40 | 3265.59 | -5339.01 | 1222.78 | -1816.17 | 169712.69 | -276009.99 | 4452.42 | -6745.20 | 3268.81 | -4539.19 | 503.18 | -619.32 |
| V894G | 251.09 | -324.08 | 1551.18 | -2653.01 | 2019.85 | -3406.65 | 4915.71 | -8830.46 | 1753.34 | -2931.26 | 206221.56 | -404176.21 | 6828.58 | -11608.24 | 3921.93 | -6160.47 | 642.21 | -874.36 |
| V902F | 149.44 | -172.24 | 1194.22 | -2156.46 | 1866.32 | -3168.90 | 3510.00 | -6333.53 | 1331.07 | -2243.23 | 179964.71 | -354834.14 | 5003.31 | -8469.47 | 934.34 | -1422.68 | 469.96 | -615.43 |
| V937E | 154.39 | -118.92 | 1434.02 | -2462.56 | 2471.36 | -4580.54 | 3924.22 | -6556.74 | 1264.21 | -1792.38 | 180934.07 | -295516.07 | 5260.25 | -8057.03 | 2711.12 | -3767.30 | 539.26 | -655.49 |
| V916F | 142.84 | -85.17 | 1010.17 | -1291.05 | 1425.86 | -1648.20 | 4171.95 | -6382.43 | 1280.42 | -1664.10 | 163406.21 | -232956.74 | 5318.32 | -6960.53 | 2476.25 | -2609.91 | 417.35 | -355.96 |
| V896G | 176.91 | -145.27 | 1272.08 | -1794.14 | 2134.31 | -3398.45 | 5035.13 | -8420.73 | 1860.12 | -2860.36 | 205631.46 | -349189.65 | 5562.85 | -7995.52 | 3423.19 | -4289.80 | 540.92 | -523.71 |
| V935F | 130.24 | -76.15 | 807.63 | -712.56 | 1236.50 | -955.41 | 2283.53 | -2098.13 | 811.90 | -581.72 | 102870.30 | -79644.98 | 2609.47 | -831.68 | 1762.13 | -998.86 | 314.11 | -158.27 |
| V916G | 209.30 | -173.44 | 1276.84 | -1312.79 | 2180.72 | -2543.85 | 4342.05 | -5083.43 | 1556.42 | -1697.44 | 169583.11 | -189957.38 | 5838.22 | -5763.36 | 3643.29 | -2892.00 | 480.89 | -287.26 |
| V792BA | 166.02 | -100.68 | 1117.49 | -1050.12 | 1931.58 | -2161.38 | 3582.78 | -3692.94 | 1226.17 | -1071.82 | 177669.25 | -199533.34 | 5240.18 | -4750.90 | 1671.60 | -793.50 | 409.86 | -193.98 |
| V894I | 173.73 | -110.13 | 1357.26 | -1449.69 | 1965.77 | -2250.00 | 3349.64 | -3432.53 | 1279.39 | -1280.73 | 149132.81 | -142612.65 | 6054.89 | -5921.08 | 2815.43 | -1999.27 | 518.87 | -280.38 |
| V896BB | 125.32 | -69.04 | 1112.70 | -1187.86 | 1656.11 | -2006.75 | 2969.28 | -3202.47 | 1241.09 | -1438.74 | 123040.69 | -137678.56 | 4681.80 | -4498.95 | 2482.19 | -1987.66 | 362.39 | -200.74 |
| V908EA | 170.27 | -122.83 | 1562.55 | -1869.21 | 2041.52 | -2678.62 | 4556.60 | -5932.82 | 1596.18 | -1751.14 | 166571.39 | -190162.85 | 4881.05 | -5402.31 | 2586.60 | -2216.24 | 455.58 | -372.69 |
| V908J | 205.35 | -79.96 | 1287.76 | -1079.19 | 2092.62 | -2177.20 | 4906.58 | -5546.57 | 1759.57 | -1510.33 | 156804.48 | -147252.65 | 5759.74 | -5043.80 | 2972.97 | -1809.19 | 522.63 | -220.56 |
| V935G | 158.47 | -101.62 | 1093.96 | -1167.80 | 1933.48 | -2366.93 | 3336.31 | -3879.29 | 1170.96 | -1152.99 | 169393.39 | -191025.76 | 4987.93 | -4406.85 | 2557.19 | -1987.22 | 446.99 | -305.63 |
| V916H | 232.25 | -149.73 | 1301.55 | -1209.60 | 1835.35 | -1835.56 | 3880.05 | -4021.32 | 1597.80 | -1594.89 | 186495.72 | -193661.09 | 5442.78 | -4224.23 | 2937.30 | -2099.16 | 454.97 | -199.53 |
| V908K | 233.69 | -185.82 | 1185.80 | -1234.44 | 2330.79 | -2797.38 | 4128.40 | -4635.23 | 1704.97 | -1967.70 | 195520.91 | -214767.48 | 5332.38 | -5276.25 | 2855.96 | -2483.77 | 475.87 | -269.59 |
| **Mean** | 178.78 | -139.73 | 1237.89 | -1536.34 | 1942.81 | -2584.14 | 3884.86 | -5211.73 | 1416.03 | -1709.69 | 168934.55 | -224936.22 | 5203.39 | -5997.21 | 2688.77 | -2628.51 | 472.19 | -383.31 |
| **SD** | 37.76 | 65.90 | 192.87 | 549.87 | 308.56 | 866.68 | 767.61 | 1847.96 | 282.14 | 604.93 | 27253.78 | 89046.26 | 896.46 | 2384.90 | 756.38 | 1416.32 | 77.05 | 210.43 |
| **Shapiro-Wilk (Normal distribution test)** | | | |  |  |  |  |  |  |  |  |  |  |  |  |  |  |  |
| **Obs Test Stat** | 0.95 | 0.87 | 0.97 | 0.92 | 0.94 | 0.97 | 0.97 | 0.97 | 0.93 | 0.94 | 0.92 | 0.94 | 0.88 | 0.94 | 0.95 | 0.90 | 0.97 | 0.87 |
| **P-value** | 0.42 | 0.02 | 0.78 | 0.16 | 0.39 | 0.81 | 0.79 | 0.76 | 0.23 | 0.40 | 0.17 | 0.37 | 0.04 | 0.40 | 0.51 | 0.08 | 0.86 | 0.03 |
| **Comparison with the general pattern** | | | | | | | | | | |  |  |  |  |  |  |  |  |
| **FULL DATASET** | **177.33** | **-126.63** | **1233.91** | **-1423.89** | **1925.15** | **-2410.45** | **3861.46** | **-4835.30** | **1424.25** | **-1623.53** | **162313.09** | **-193597.24** | **5194.88** | **-5529.72** | **2642.74** | **-2289.72** | **464.58** | **-326.20** |
| **Obs Test Stat** | 0.15 | N/A | 0.08 | -0.82 | 0.23 | -0.80 | 0.12 | -0.81 | -0.12 | -0.57 | 0.97 | -1.41 | N/A | -0.78 | 0.24 | -0.96 | 0.39 | -1.09 |
| **P-value** | 0.88 | N/A | 0.94 | 0.43 | 0.82 | 0.44 | 0.90 | 0.43 | 0.91 | 0.58 | 0.35 | 0.18 | N/A | 0.45 | 0.81 | 0.35 | 0.70 | 0.29 |

| **TABLE S3.d** Individual growth pattern of segment inertia (y axis) represented by the linear regression model (y=a*age+b) in males | | | | | | | | | | | | | | | | | | |
| --- | --- | --- | --- | --- | --- | --- | --- | --- | --- | --- | --- | --- | --- | --- | --- | --- | --- | --- |
|  | Hand |  | Forearm | | Upper arm | | Thigh |  | Shank |  | Trunk |  | Head |  | Tail |  | Foot |  |
|  | a | b | a | b | a | b | a | b | a | b | a | b | a | b | a | b | a | b |
| V908H | 170.66 | -204.37 | 1238.96 | -1947.34 | 1997.52 | -3436.78 | 3430.57 | -5645.97 | 1233.78 | -1837.18 | 169041.58 | -274879.63 | 4480.64 | -6788.02 | 3268.81 | -4539.19 | 487.37 | -599.18 |
| V894G | 240.74 | -309.82 | 1548.80 | -2649.77 | 2055.41 | -3472.63 | 5215.28 | -9483.00 | 1770.89 | -2967.22 | 205101.17 | -401862.06 | 6954.27 | -11868.16 | 3921.93 | -6160.47 | 622.45 | -846.24 |
| V902F | 143.64 | -165.96 | 1191.14 | -2150.92 | 1897.39 | -3229.99 | 3646.67 | -6606.15 | 1339.84 | -2260.67 | 178402.82 | -351089.56 | 5079.35 | -8615.64 | 934.34 | -1422.68 | 453.69 | -590.58 |
| V937E | 147.02 | -110.74 | 1435.93 | -2467.64 | 2531.62 | -4705.20 | 4111.88 | -6933.33 | 1274.61 | -1811.72 | 179456.67 | -292458.94 | 5345.24 | -8219.24 | 2711.12 | -3767.30 | 526.56 | -642.73 |
| V916F | 135.04 | -76.93 | 1008.47 | -1289.38 | 1452.48 | -1688.40 | 4425.37 | -6870.60 | 1293.40 | -1686.95 | 161724.38 | -229927.96 | 5399.48 | -7063.53 | 2476.25 | -2609.91 | 403.17 | -345.22 |
| V896G | 169.82 | -139.21 | 1269.29 | -1787.92 | 2172.82 | -3460.00 | 5288.70 | -8885.04 | 1873.97 | -2883.36 | 204400.84 | -346723.27 | 5609.20 | -8059.04 | 3423.19 | -4289.80 | 523.35 | -508.30 |
| V935F | 124.86 | -72.82 | 806.34 | -710.98 | 1249.87 | -963.85 | 2365.79 | -2187.75 | 816.38 | -585.76 | 102440.52 | -79148.45 | 2646.98 | -869.48 | 1762.13 | -998.86 | 306.29 | -154.36 |
| V916G | 199.26 | -164.06 | 1273.79 | -1309.96 | 2240.13 | -2626.06 | 4553.79 | -5363.19 | 1569.11 | -1714.28 | 168404.66 | -188131.27 | 5902.14 | -5839.55 | 3643.29 | -2892.00 | 464.74 | -273.84 |
| V792BA | 159.43 | -96.78 | 1116.56 | -1050.06 | 1969.12 | -2209.06 | 3735.49 | -3864.38 | 1232.96 | -1079.43 | 176726.40 | -198109.39 | 5320.42 | -4845.29 | 1671.60 | -793.50 | 399.56 | -189.00 |
| V894I | 165.54 | -104.12 | 1356.14 | -1449.66 | 2007.13 | -2303.50 | 3533.28 | -3634.78 | 1293.06 | -1298.77 | 148188.70 | -141355.23 | 6166.82 | -6074.70 | 2815.43 | -1999.27 | 502.91 | -268.81 |
| V896BB | 119.36 | -65.25 | 1111.88 | -1188.30 | 1694.72 | -2065.02 | 3118.17 | -3386.58 | 1249.90 | -1452.24 | 122049.32 | -136462.01 | 4751.03 | -4577.86 | 2482.19 | -1987.66 | 351.01 | -193.12 |
| V908EA | 163.65 | -118.61 | 1558.94 | -1866.09 | 2081.48 | -2741.20 | 4826.28 | -6336.37 | 1608.44 | -1769.62 | 165340.66 | -188374.07 | 4938.74 | -5464.27 | 2586.60 | -2216.24 | 444.62 | -366.52 |
| V908J | 197.89 | -79.44 | 1286.17 | -1078.90 | 2132.94 | -2221.05 | 5219.24 | -5968.97 | 1774.30 | -1529.86 | 155200.48 | -145420.06 | 5827.65 | -5119.65 | 2972.97 | -1809.19 | 507.22 | -215.86 |
| V935G | 151.50 | -96.64 | 1093.46 | -1168.42 | 1969.77 | -2415.51 | 3528.48 | -4132.66 | 1179.26 | -1162.93 | 167900.39 | -188883.82 | 5025.61 | -4454.85 | 2557.19 | -1987.22 | 435.21 | -297.90 |
| V916H | 222.83 | -143.81 | 1298.50 | -1207.70 | 1866.40 | -1869.24 | 4060.22 | -4240.17 | 1611.47 | -1612.06 | 185336.73 | -192135.48 | 5513.13 | -4307.30 | 2937.30 | -2099.16 | 439.73 | -189.95 |
| V908K | 225.14 | -179.84 | 1183.53 | -1233.37 | 2379.48 | -2860.77 | 4312.68 | -4856.30 | 1719.17 | -1988.60 | 193692.92 | -212169.26 | 5371.01 | -5305.18 | 2855.96 | -2483.77 | 464.22 | -263.56 |
| **Mean** | **171.02** | **-133.03** | **1236.12** | **-1534.78** | **1981.14** | **-2641.77** | **4085.74** | **-5524.70** | **1427.54** | **-1727.54** | **167713.01** | **-222945.65** | **5270.73** | **-6091.99** | **2688.77** | **-2628.51** | **458.26** | **-371.57** |
| **SD** | **36.53** | **62.80** | **192.64** | **549.46** | **318.52** | **888.34** | **824.55** | **1981.45** | **285.00** | **611.14** | **27067.88** | **88470.25** | **914.31** | **2429.89** | **756.38** | **1416.32** | **74.54** | **204.09** |
| **Shapiro-Wilk (Normal distribution test)** | | | |  |  |  |  |  |  |  |  |  |  |  |  |  |  |  |
| **Obs Test Stat** | 0.94 | 0.87 | 0.97 | 0.92 | 0.95 | 0.97 | 0.96 | 0.97 | 0.93 | 0.95 | 0.92 | 0.94 | 0.88 | 0.94 | 0.95 | 0.90 | 0.97 | 0.87 |
| **P-value** | 0.37 | 0.02 | 0.77 | 0.15 | 0.42 | 0.80 | 0.68 | 0.79 | 0.24 | 0.41 | 0.19 | 0.36 | 0.04 | 0.39 | 0.51 | 0.08 | 0.87 | 0.03 |
| **Comparison with the general pattern** | | | | | |  |  |  |  |  |  |  |  |  |  |  |  |  |
| **FULL DATASET** | **169.77** | **-121.02** | **1232.18** | **-1422.69** | **1963.93** | **-2466.18** | **4065.20** | **-5134.29** | **1435.88** | **-1640.67** | **161090.04** | **-191720.68** | **5263.64** | **-5621.16** | **2642.74** | **-2289.72** | **450.86** | **-316.19** |
| **Obs Test Stat** | 0.14 | N/A | 0.08 | -0.82 | 0.22 | -0.79 | 0.10 | -0.79 | -0.12 | -0.57 | 0.98 | -1.41 | N/A | -0.78 | 0.24 | -0.96 | 0.40 | -1.09 |
| **P-value** | 0.89 | N/A | 0.94 | 0.43 | 0.83 | 0.44 | 0.92 | 0.44 | 0.91 | 0.58 | 0.34 | 0.18 | N/A | 0.45 | 0.81 | 0.35 | 0.70 | 0.29 |

| **TABLE S4.** Individual linear regression models (RMA) in female baboons | | | | | | | | | | |
| --- | --- | --- | --- | --- | --- | --- | --- | --- | --- | --- |
|  | Forelimb x Hindlimb | | | | | |  |  |  |  |
|  | Mass (kg) | | Length (mm) | | NPP (s) | | I (kg.mm⁻²) | | COM (mm) | |
|  | a | b | a | b | a | b | a | b | a | b |
| V913G | 1.36 | 0.07 | 0.92 | 77.00 | 0.81 | 0.22 | 1.09 | 6849.06 | 0.75 | 22.91 |
| V935E | 1.86 | -0.04 | 1.03 | 41.91 | 0.86 | 0.17 | 1.43 | 4743.94 | 0.74 | 27.46 |
| V936F | 1.75 | 0.07 | 1.00 | 53.15 | 1.01 | -0.03 | 1.54 | 3687.94 | 0.97 | -13.85 |
| V915G | 1.94 | -0.04 | 1.05 | 39.06 | 0.84 | 0.20 | 1.63 | 1006.29 | 0.79 | 20.86 |
| V936G | 1.77 | 0.00 | 1.05 | 22.34 | 0.96 | 0.06 | 1.64 | 365.85 | 0.93 | -0.01 |
| V902G | 1.92 | -0.05 | 1.14 | -8.43 | 0.93 | 0.08 | 1.67 | -1759.33 | 0.87 | 7.29 |
| V908I | 2.20 | -0.14 | 1.06 | 28.45 | 0.81 | 0.24 | 1.77 | -1545.52 | 0.74 | 31.45 |
| V894H | 2.07 | -0.15 | 1.19 | -28.59 | 0.89 | 0.12 | 1.59 | -4275.73 | 0.77 | 18.64 |
| V792BB | 1.76 | 0.00 | 1.09 | 15.40 | 0.98 | 0.03 | 1.62 | -928.59 | 0.96 | -8.45 |
| V916DA | 1.79 | -0.01 | 1.15 | -2.36 | 0.95 | 0.06 | 1.50 | 2320.13 | 0.82 | 14.93 |
| V916I | 1.94 | -0.06 | 1.07 | 23.73 | 0.87 | 0.17 | 1.68 | 9.16 | 0.78 | 24.86 |
| V993EB | 1.66 | 0.03 | 1.02 | 37.47 | 0.82 | 0.22 | 1.28 | 5874.37 | 0.71 | 30.47 |
| V936H | 1.75 | -0.01 | 1.05 | 25.37 | 0.98 | 0.01 | 1.53 | 482.36 | 0.91 | 0.11 |
| V908GA | 1.68 | 0.02 | 1.08 | 20.95 | 0.90 | 0.11 | 1.38 | 3024.32 | 0.81 | 12.88 |
| **Mean** | **1.82** | **-0.02** | **1.06** | **24.68** | **0.90** | **0.12** | **1.52** | **1418.16** | **0.82** | **13.54** |
| **SD** | **0.20** | **0.07** | **0.07** | **26.31** | **0.07** | **0.09** | **0.18** | **3161.11** | **0.09** | **14.51** |
| **Shapiro-Wilk (Normal distribution test)** | | | |  |  |  |  |  |  |  |
| **Obs Test Stat** | 0.95 | 0.92 | 0.96 | 0.97 | 0.94 | 0.95 | 0.92 | 0.98 | 0.91 | 0.94 |
| **P-value** | 0.55 | 0.21 | 0.61 | 0.85 | 0.38 | 0.55 | 0.20 | 0.96 | 0.14 | 0.35 |
| **Comparison with the general pattern** | | |  |  |  |  |  |  |  |  |
| **FULL DATASET** | **1.81** | **-0.02** | **1.08** | **19.50** | **0.90** | **0.12** | **1.50** | **1626.86** | **0.81** | **15.30** |
| **Obs Test Stat** | 0.17 | -0.09 | -0.86 | 0.74 | 0.04 | -0.06 | 0.52 | -0.25 | 0.59 | -0.45 |
| **P-value** | 0.87 | 0.93 | 0.41 | 0.47 | 0.97 | 0.95 | 0.61 | 0.81 | 0.56 | 0.66 |

| **TABLE S5.** Individual linear regression models (RMA) in male baboons | | | | | | | | | | |
| --- | --- | --- | --- | --- | --- | --- | --- | --- | --- | --- |
|  | Forelimb x Hindlimb | |  |  |  |  |  |  |  |  |
|  | Mass (kg) | | Length (mm) | | NPP (s) | | I (kg.mm⁻²) | | COM (mm) | |
|  | a | b | a | b | a | b | a | b | a | b |
| V908H | 1.53 | 0.04 | 1.10 | 6.83 | 0.89 | 0.13 | 1.24 | 8107.63 | 0.81 | 16.58 |
| V894G | 1.85 | -0.03 | 1.15 | -18.68 | 1.03 | -0.08 | 1.52 | -3150.16 | 0.92 | -11.36 |
| V902F | 1.75 | -0.03 | 1.08 | 12.80 | 0.93 | 0.08 | 1.40 | 3458.54 | 0.79 | 18.78 |
| V937E | 1.58 | 0.03 | 1.04 | 37.57 | 0.85 | 0.19 | 1.22 | 13370.56 | 0.72 | 35.24 |
| V916F | 2.21 | -0.25 | 1.11 | 9.31 | 0.78 | 0.28 | 1.62 | -1992.89 | 0.68 | 40.37 |
| V896G | 1.97 | -0.09 | 1.14 | -4.88 | 1.00 | 0.00 | 1.79 | -6936.13 | 0.93 | -2.84 |
| V935F | 1.72 | -0.05 | 1.04 | 23.10 | 0.85 | 0.16 | 1.24 | 2352.34 | 0.71 | 28.10 |
| V916G | 1.71 | -0.03 | 1.10 | 6.38 | 0.95 | 0.05 | 1.41 | 2986.39 | 0.89 | 3.32 |
| V792BA | 1.64 | 0.05 | 1.08 | 18.17 | 0.89 | 0.13 | 1.33 | 6179.17 | 0.81 | 13.22 |
| V894I | 1.63 | 0.04 | 1.07 | 19.28 | 0.89 | 0.12 | 1.29 | 5202.53 | 0.75 | 22.02 |
| V896BB | 1.67 | 0.01 | 1.07 | 18.43 | 0.88 | 0.14 | 1.39 | 1787.44 | 0.76 | 21.78 |
| V908EA | 1.87 | -0.01 | 1.04 | 31.87 | 0.84 | 0.16 | 1.30 | 4313.59 | 0.72 | 19.80 |
| V908J | 1.99 | -0.10 | 1.12 | 3.31 | 0.87 | 0.14 | 1.55 | -2588.18 | 0.74 | 24.11 |
| V935G | 1.69 | 0.02 | 1.04 | 34.56 | 0.86 | 0.16 | 1.25 | 5401.64 | 0.71 | 26.30 |
| V916H | 1.81 | -0.08 | 1.09 | 9.03 | 0.89 | 0.11 | 1.36 | 1137.59 | 0.79 | 16.04 |
| V908K | 1.62 | 0.05 | 1.09 | 10.04 | 0.95 | 0.05 | 1.42 | 1212.66 | 0.85 | 6.49 |
| **Mean** | **1.77** | **-0.03** | **1.08** | **13.57** | **0.90** | **0.11** | **1.39** | **2552.67** | **0.79** | **17.37** |
| **SD** | **0.18** | **0.08** | **0.03** | **14.50** | **0.06** | **0.08** | **0.16** | **4860.44** | **0.08** | **13.43** |
| **Shapiro-Wilk (Normal distribution test)** | | | |  |  |  |  |  |  |  |
| **Obs Test Stat** | 0.92 | 0.85 | 0.95 | 0.97 | 0.95 | 0.94 | 0.90 | 0.98 | 0.93 | 0.97 |
| **P-value** | 0.18 | 0.02 | 0.50 | 0.73 | 0.44 | 0.36 | 0.08 | 0.93 | 0.25 | 0.82 |
| **Comparison with the general pattern** | | |  |  |  |  |  |  |  |  |
| **FULL DATASET** | **1.78** | **-0.03** | **1.09** | **13.39** | **0.90** | **0.11** | **1.41** | **1954.50** | **0.79** | **16.32** |
| **Obs Test Stat** | -0.33 | N/A | -0.67 | 0.05 | 0.20 | 0.18 | -0.36 | 0.49 | -0.20 | 0.31 |
| **P-value** | 0.74 | N/A | 0.51 | 0.96 | 0.84 | 0.86 | 0.72 | 0.63 | 0.84 | 0.76 |
